# Immune regulatory cytokines in seminal plasma of healthy men: A scoping review and analysis of variance

**DOI:** 10.1111/andr.13424

**Published:** 2023-04-10

**Authors:** Hannah E. Lyons, Bridget M. Arman, Sarah A. Robertson, David J. Sharkey

**Affiliations:** ^1^ Robinson Research Institute and School of Biomedicine University of Adelaide Adelaide South Australia Australia; ^2^ Department of Obstetrics and Gynaecology University of Melbourne Parkville Melbourne Australia

**Keywords:** chemokines, cytokines, male fertility, semen analysis, seminal plasma

## Abstract

**Objective:**

Seminal plasma cytokines are associated with fertility and reproductive health, but progressing their clinical utility is hampered by absence of reference data on concentration ranges of relevant cytokines in healthy men. We employed a systematic approach to assemble current evidence on the concentrations of immune regulatory cytokines present in seminal plasma (SP) of normozoospermic and/or fertile men and evaluated the impact of different platform methodologies for cytokine quantification.

**Evidence review:**

A systematic literature search was performed utilising PubMed, Web of Science and Scopus. Databases were searched from inception until 30th June 2022 inclusive, using combinations of keywords pertaining to seminal fluid and cytokines, and was restricted to human participants. Original data with values reported as concentration of specific cytokines in SP of men clearly defined as fertile or normozoospermic were extracted from studies written in English.

**Results:**

A total of 3769 publications were initially identified, of which 118 fulfilled the eligibility criteria for inclusion. A total of 51 individual cytokines are detectable in SP of healthy men. The number of studies reporting on each cytokine range from 1 to >20. The reported concentrations for many cytokines linked with fertility status, including IL6, CXCL8/IL8, and TNFA, are highly variable between published studies. This is associated with the different immunoassay methodologies utilised and may be exacerbated by a lack of validation of assays to ensure suitability for SP assessment. Due to the large variation between studies, accurate reference ranges for healthy men cannot be determined from the published data.

**Conclusions:**

The concentrations of cytokines and chemokines detected in SP is inconsistent and highly variable between studies and cohorts, limiting current capacity to define reference ranges for cytokine concentrations in fertile men. The lack of standardisation in methods used to process and store SP, and variation in platforms used to evaluate cytokine abundance, are factors contributing to the observed heterogeneity. To progress the clinical utility of SP cytokine analysis will require standardisation and validation of methodologies so that reference ranges for healthy fertile men can be defined.

## 1 INTRODUCTION

Assessment of male fertility potential is currently defined based on parameters of semen quality including sperm concentration, motility, morphology, volume, pH and viscosity.^1^ Sperm quality can vary substantially between men and even within individuals because of age, period of ejaculatory abstinence, diet and nutrition, microbiome and infection, occupation and environmental exposures, as well as genetics.^2^, ^3^ Defining fertility based on sperm quality is problematic, because having sperm parameters at or above arbitrary thresholds does not guarantee capacity to achieve unassisted conception,^4^ as recognised in revised World Health Organization (WHO) guidelines for the assessment of semen.^1^ There is growing recognition that improved prognostic value may be achieved when semen analysis results are evaluated in conjunction with several additional parameters. Immune regulatory cytokines in the seminal plasma (SP) fraction of the ejaculate are potentially informative additional biomarkers of male fertility, but standardised protocols for their evaluation are not yet available.

Cytokines are a large and diverse family of small proteins secreted by several cell types that have specific activity in immunomodulatory and developmental processes, including cellular differentiation, activation, and migration. Seminal fluid (whole semen) contains a diverse array of cytokines, chemokines, and growth factors, mostly in soluble form in the SP fraction. Cytokines are central to regulation of most reproductive tissues and processes, although understanding of their functional significance in SP and relevance to male fertility and fecundity is still evolving.

There is an extensive literature on the identification and quantification of numerous cytokines and chemokines in SP. Many hundreds of studies have investigated potential relationships between SP cytokines and sperm parameters, genitourinary pathophysiology, and/or fertility and pregnancy outcomes. Most studies examine the relationship between cytokine concentration in SP and parameters of sperm quality, including sperm concentration,^5^, ^6^, ^7^, ^8^ motility,^5^, ^7^, ^8^, ^9^, ^10^ morphology,^5^, ^6^ and viscosity.^11^ The concentration and total abundance of cytokines within ejaculates appears to be highly variable between men and can fluctuate within individuals over time.^12^, ^13^ An increased abundance of certain cytokines has been associated with the presence of high numbers of leukocytes in the ejaculate, referred to as leukocytospermia, which may indicate male genital tract infection with bacterial, viral, or parasitic infections that increase the likelihood of infertility. Other cytokines such as interleukin CXCL8/(IL)8 are associated with male genital tract inflammation caused by either infection or sterile injury.^8^, ^14^, ^15^, ^16^, ^17^, ^18^, ^19^, ^20^, ^21^, ^22^, ^23^, ^24^, ^25^, ^26^

Several SP cytokines have physiological roles in supporting sperm function and fertilising capacity. Animal studies show that cytokines in seminal fluid also promote reproductive success through eliciting an immune response in the female reproductive tract after intromission. Seminal plasma can substantially influence embryo development, endometrial remodelling and the likelihood of successful implantation, in turn affecting placental development and offspring health.^27^, ^28^ In women, a beneficial effect of previous seminal fluid exposure on fertility and pregnancy outcome is reported.^29^, ^30^, ^31^ Importantly, SP cytokines can exert a pro‐inflammatory or an anti‐inflammatory effect, implying the balance of immune regulatory factors within SP is important for the strength and quality of the female immune response.

The most recent edition of the WHO guidelines proposes that analysis of specific cytokines in SP may be useful in diagnostic assessment of male infertility, particularly inflammatory states of the male genital tract.^1^ However, advancing the clinical application of SP cytokine analysis in fertility assessment is hampered by the lack of standardized approaches to analysis. In particular, there is no consensus definition of the normal ranges of these cytokines within normozoospermic and proven fertile men, or understanding of relationships with age, ethnicity, or other relevant factors. This lack of reference intervals for SP cytokines limits investigation of their relationships with sperm parameters, fertility, and other clinical factors and determinants. We hypothesised that the considerable variation between published studies in the reported concentrations and abundance of individual cytokines may be because of differences in the technical platforms used to quantify cytokine abundance, as well as variables such as subject characteristics and methodology for SP preparation and storage. Here, we survey the biomedical literature to synthesise a current understanding of the range and variability in SP cytokine concentrations reported for normozoospermic and proven fertile men, and to relate these to the platform technologies by which cytokines are analysed. The outcomes will help inform development of consensus approaches for future investigation of the clinical utility of SP cytokine analysis.

## 2 METHODS

### 2.1 Information sources and search terms

A systematic search of scientific databases PubMed, Scopus and Web of Science was performed to identify studies that quantified cytokines in SP. The search terms combined keywords pertaining to semen including SP, seminal fluid, ejaculate, and semen, with cytokine‐associated keywords including interleukin, growth factor, cluster of differentiation, defensin, cytokine, chemokine, monokine, lymphokine, prostaglandin, interferon, tumour necrosis factor, and human leukocyte antigen. Studies were restricted to those including human participants by using search terms ‘human’, ‘men’, ‘donor’, ‘participant’ and ‘patient’. The full search strategy is provided in Table S1. Databases were searched from publication dates spanning inception until the 30th of June 2022 inclusive to identify all relevant papers.

### 2.2 Eligibility criteria

The literature search was limited to the English language and strictly human subjects. Studies without original data, such as reviews, were excluded, as were conference abstracts, theses/dissertations, opinion pieces, and editorial letters. Studies that did not report the concentration of SP cytokines were excluded, including any in which data were presented in figures without clearly legible axes. Studies that did not include discernible data sets from healthy men that were normozoospermic and/or proven fertile, were excluded. Studies with ambiguous cohort status, that did not report fertility status or provide details of semen analysis of participants, were excluded. Studies that included men with chronic medical conditions, significant smoking habits, or evidence of alcohol or drug abuse, leukocytospermia, azoospermia (absence of spermatozoa in the ejaculate), asthenozoospermia (reduced sperm motility), vasectomy, recent history of urogenital disease or sexually transmitted infections, or testicular diseases such as varicocoele, hydrocele, and orchitis were all excluded, unless data sets from men with these factors were clearly discernible from data sets from unaffected healthy men, in which case only the latter data sets were extracted.

Studies that did not evaluate SP but evaluated only whole semen or spermatozoa‐associated cytokines were excluded. In cases where the investigated fraction of seminal fluid was potentially ambiguous (e.g., when the term ‘seminal fluid’ was used rather than ‘SP’), the studies were included, after confirmation that seminal plasma was indeed utilized.

### 2.3 Study selection and data extraction

**FIGURE 1:**
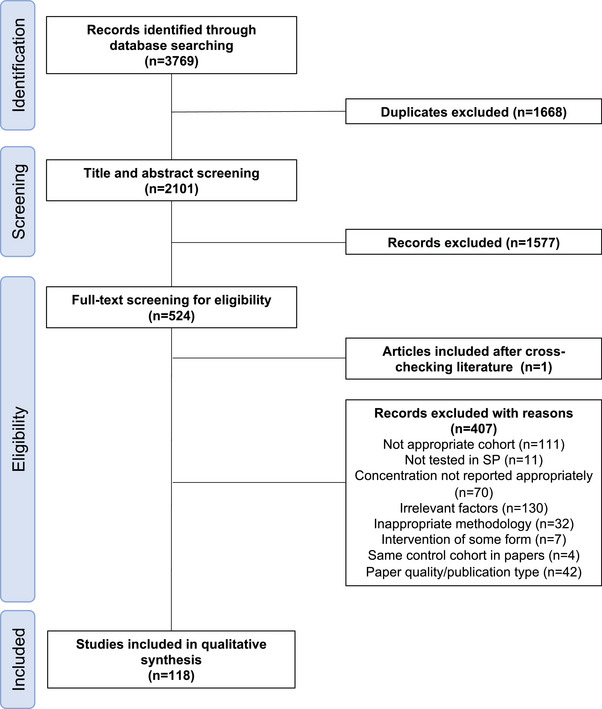
Preferred reporting items for systematic reviews and meta‐analyses (PRISMA) flow diagram (adapted from Moher et al. 2009^33^).

All articles were imported directly from databases to Endnote X9 software (Clarivate Analytics, Philadelphia USA). After removal of duplicates [method described by Bramer et al. 2016],^32^ two reviewers (H. E. L. and B. M. A.) independently screened the title, abstract and full text of the papers. Queries about appropriateness of paper inclusion were resolved in consultation with additional reviewers (S. A. R. and D. J. S.). Two reviewers (H. E. L. and B. M. A.) performed data extraction on included studies, extracting study demographics (authors, year of publication, country, number and ages of participants) and outcome data (SP cytokine concentrations, and methodologies). H. E. L. analysed the extracted data and synthesised the results. A PRISMA flow diagram outlining study selection, number of papers included within review, numbers of papers excluded from review and reasons for exclusion are included in Figure 1 (adapted from Moher et al. 2009).^33^ The data extracted from these 118 studies are provided in Supporting Dataset 1 in Microsoft Excel (.xslx) format (Supporting_Dataset_1.xlsx: doi:10.25909/22186528). When concentrations were reported in units other than pg/mL or ng/mL, values were converted to these concentrations using the molecular weights of 15.3 kDa for IL2, 55 kDa for sIL2R, 25 kDa for IFNG and 17 kDa for TNFA.

### 2.4 Risk of bias in individual studies

In considering the risk of bias within individual studies we evaluated the cohort sizes included in the studied populations, the methodology that was used to prepare and store SP samples, the analytical techniques utilised for cytokine quantification, as well as known exposures and risk factors in studied populations (i.e., occupational, environmental, medication use etc.).

### 2.5 Synthesis of results

Seminal plasma factors were categorised as either cytokines or chemokines, and then further into cytokine families including interleukins (ILs), interferons (IFNs), tumour necrosis factors (TNFs), transforming growth factor beta (TGFB) superfamily, colony stimulating factors (CSFs), C‐X‐C chemokine ligands, and CC chemokine ligands.

### 2.6 Additional analysis

It was not possible to perform a meta‐analysis because of heterogeneity of the data resulting from differences in the descriptive statistics reported, the platform techniques used for cytokine quantification, and demographic differences in the cohorts investigated. IBM SPSS Statistics version 28.0 (IBM Corp., Armonk, NY, USA) for Windows was used for performing mixed model analysis. A linear mixed model analysis was used to evaluate the relative effects of assay platform used, fertility status (normozoospermic or fertile), SP storage conditions (either < −70°C, > −70°C, or unspecified), and method of SP preparation (centrifugation at ≥ 13,000 x g for at least 15 min, a different method, or unspecified) on reported cytokine concentration. Where an effect of assay platform was identified, pair‐wise comparisons between the different assay platforms was assessed and significant differences noted. Linear mixed model analysis was performed only on cytokines where the number of studies reporting on the concentration of the specific cytokine of interest was greater than 5, because of there being insufficient observations compared to the tested parameters. Further, for mixed model analysis, where only two methodologies were utilised to measure a cytokine, the criteria of there being at least two studies using each methodology was employed. GraphPad Prism version 9 for Windows (GraphPad Software, San Diego, California USA, www.graphpad.com) was used to generate bubble plots and calculate coefficients of variance. Datasets were tested for outliers using the ROUT test (Q = 0.01%) and data with these identified outliers removed was used to calculate a second coefficient of variance for each reported cytokine. Variance was then categorised as being either low (CV < 75%), moderate (CV ≥ 75 and < 150%) or high (CV > 150%). For all statistical tests, differences were considered significant when *p* < 0.05.

## 3 RESULTS

### 3.1 Study selection and characteristics

A total of 3769 references were retrieved (PubMed: 1302, Scopus: 1510, and Web of Science: 1510). After the removal of 1668 duplicate articles, 2101 articles were eligible for title and abstract screening. During this phase of screening, a further 1577 articles were excluded, leaving 524 articles eligible for full‐text screening. One additional study was discovered in the reference lists of studies undergoing full‐text screening. Next, a further 407 articles were excluded for reasons including inappropriate study cohorts or methodology, or cytokine concentrations not being reported. In total, 118 studies were found that fulfilled the inclusion criteria.

### 3.2 Description of participants

A description of participant characteristics is provided in Supporting Dataset 1. All men were aged between 18−66 years (with most study cohorts within reproductive age (18–45 years)), with no reported significant history of smoking, alcohol or drug use, with normal sperm parameters and/or proven fertility, and in good health with no chronic disease, testicular disease, urogenital disease, or sexually transmitted infections.

### 3.3 Description of interventions

The studies included in this review were limited to those that included defined data sets for normozoospermic and/or proven fertile men. Where other participants were included as separate groups, data from the other groups were not extracted. None of the men in any of the included studies underwent any intervention. Many of the included studies were in an assisted reproductive technology (ART) clinic setting, wherein men were undergoing routine fertility assessment or were fertile male partners of women experiencing infertility. Studies utilising participants attending an ART clinic were included only when semen analysis was reported and classified as normozoospermic.

### 3.4 Outcomes

The systematic review identified 118 studies, published between 1993 and 30th June 2022 inclusive, that reported cytokine concentrations in SP and fulfilled the inclusion criteria. All studies quantified the abundance of one or usually several cytokines in SP. Data on a total of 51 different cytokines were reported. There was wide variation in the identity of cytokines measured and reported in each study, and consequently in the number of studies available for each cytokine (Table 1).

**TABLE 1:** Summary of the cytokines that were investigated, number of studies, year of publication and total number of participants

Factor	Number of studies	Years of publication	Total number of participants
Interleukins (ILs)			
IL1A	6	1996–2021	272
IL1B	35	1994–2022	1366^†^
IL1RA	4	1996–2016	41
IL2	19	1993–2021	768
sIL2R	8	1994–2016	192
IL4	17	1996–2021	635
IL5	9	2007–2020	580
IL6	64	1994–2022	2966^†^
IL7	5	2007–2020	304
IL9	1	2016	10
IL10	24	1996–2022	1186
IL11	5	1996–2011	110
IL12	17	1998–2022	885
IL13	8	2007–2020	614
IL15	1	2016	10
IL16	2	2016–2022	21
IL17	13	2007–2021	469
IL18	9	2006–2022	233
IL23	2	2011–2014	45
IL33	1	2021	11
IL37	1	2017	75
LIF	2	1996–2016	13
**Transforming growth factor beta family (TGFB)**			
TGFB1 (bioactive)	11	1996–2021	385^†^
TGFB1 (total)	12	1996–2022	237^†^
TGFB2 (bioactive)	5	1996–2016	41^†^
TGFB2 (total)	6	1996–2022	76^†^
TGFB3 (bioactive)	3	2012–2016	35^†^
TGFB3 (total)	5	2012–2022	61^†^
Activin A	4	1998–2016	67^†^
Inhibin B	1	1998	37
FST	2	2015–2016	15^†^
GDF‐15	1	2010	19
**Interferons (IFNs)**			
IFNA	3	1998–2021	146
IFNG	26	1996–2021	1120
**Tumour necrosis factors (TNFs)**			
TNFA	41	1993–2021	2142
TNFB	2	1996–2016	13
TRAIL	3	2014–2016	60
**Colony stimulating factors (CSFs)**			
CSF1	2	2002–2016	63
CSF2	8	1996–2020	479^†^
CSF3	9	2002–2020	563^†^
**CC chemokine ligands (CCL)**			
CCL2	8	1995–2020	548
CCL3	3	1996–2016	95
CCL4	6	2007–2020	539
CCL5	3	2000–2016	121
CCL7	1	2016	10
CCL11	2	2010–2016	26
CCL27	1	2016	10
**C‐X‐C chemokine ligands (CXCL)**			
CXCL1	2	1995–2016	21
CXCL5	1	2017	60
CXCL6	1	2008	14
CXCL8	40	1993–2022	1501^†^
CXCL9	1	2008	14
CXCL10	1	2008	14
CXCL11	1	2008	14
CXCL12	2	2007‐2016	69

^†^ Studies represented as *n* = 1 because of pooling of SP samples from multiple men.

Many studies assessed relationships between cytokine concentrations and factors including fertility status, sperm parameters, reactive oxygen species, disease status, infection (HIV, chronic prostatitis, urogenital infection), and/or lifestyle factors [body mass index, alcohol intake, and smoking status], but it is beyond the scope of this review to report those data in a systematic manner, other than to comment on the potential physiological significance of each cytokine. There were considerable differences between studies in terms of description of participant characteristics, methodology for SP preparation and storage, as well as the platform for cytokine quantification.

#### 3.4.1 ILs

The interleukins are the largest of the cytokine families, comprising more than 60 members that are widely expressed in many cell types. They play key roles in immune regulation through pro‐ and anti‐inflammatory effects, and by supporting the proliferation and differentiation of different leukocyte subsets. Interleukins can be categorized into two families: the interleukin 1 family, and the common gamma chain family.

Interleukin 1 alpha (IL1A) is reported to be positively associated with accessory sex gland infection^22^ and leukocytospermia,^22^, ^34^ but appears to be not different in men with prostatitis or HIV infection,^35^, ^36^ or men with abnormal sperm parameters.^22^, ^34^ IL1A was evaluated in SP of normozoospermic and/or fertile men in six eligible studies published between 1996 and 2021,^22^, ^34^, ^35^, ^36^, ^37^, ^38^ with a combined total of 272 participants (Table 1). Five studies utilized ELISA methodology to quantify IL1A and one study utilised a microbead assay. Low variation in IL1A levels between studies was observed (CV = 69%), with a median (range) = 18.8 (6–50) pg/mL (Figure S1A, Table 2). There were insufficient studies available for analysis of effects of assay platform.

**TABLE 2:** Analysis of degree of variance between studies for each cytokine, and the effect of assay platform

		All Data				Outliers Removed			
Cytokine	Units	Mean ± SEM	Median (range)	CV	Variance	Mean ± SEM	Median (range)	CV	Variance
IL1A	pg/mL	22.4 ± 6.3	18.8 (6–50)	69%	Low	N/A	N/A	N/A	N/A
IL1B	pg/mL	245.2 ± 11.7	2.3 (0.5–391)	280%	High	10.6 ± 2.3	1.7 (0.5–27.5)	213%	High
IL1RA	pg/mL	219 ± 52.7	204 (120–349)	48%	Low	N/A	N/A	N/A	N/A
IL2	pg/mL	397 ± 255	0.8 (0.02–3963)	294%	High	5.2 ± 2.5	0.4 (0.02–34.3)	201%	High
sIL2R	pg/mL	2175 ± 831	1549 (21.2–5956)	101%	Moderate	N/A	N/A	N/A	N/A
IL4	pg/mL	3.1 ± 1.3	0.1 (0.08–18)	174%	High	0.9 ± 0.4	0.1 (0.08–5)	173%	High
IL5	pg/mL	35.8 ± 3.5	33.2 (16.5–57.1)	36%	Low	N/A	N/A	N/A	N/A
IL6	pg/mL	21.4 ± 2.3	15.9 (0.7–118)	93%	Moderate	17.7 ± 1.4	15.0 (0.7–60.2)	67%	Low
IL7	pg/mL	649 ± 46.8	610 (507–850)	20%	Low	N/A	N/A	N/A	N/A
IL10	pg/mL	25.7 ± 12.9	1.6 (0.1–357)	280%	High	2.5 ± 0.5	1.4 (0.1–10)	105%	Moderate
IL11	pg/mL	2025 ± 1307	51.8 (9–6472)	144%	Moderate	N/A	N/A	N/A	N/A
IL12	pg/mL	10.9 ± 4.9	3 (0.1–90)	212%	High	3 ± 0.5	2.8 (0.1–11)	78%	Moderate
IL13	pg/mL	10.2 ± 6.7	0.4 (0.3–48.8)	307%	High	0.4 ± 0.02	0.4 (0.3‐0.6)	21%	Low
IL17	pg/mL	19.6 ± 8.0	8 (2.2–114)	158%	High	8.6 ± 1.7	7.5 (2.2–26.8)	71%	Low
IL18	pg/mL	198 ± 48.8	170 (2.2–456)	78%	Moderate	N/A	N/A	N/A	N/A
TGFB1 (B)	pg/mL	1773 ± 774	1100 (200–9200)	145%	Moderate	1030 ± 241	850 (200–2300)	74%	Low
TGFB1 (T)	ng/mL	168 ± 39	121 (71–554)	80%	Moderate	N/A	N/A	N/A	N/A
TGFB2 (B)	pg/mL	175.6 ± 53.3	220 (8–300)	125%	Moderate	N/A	N/A	N/A	N/A
TGFB2 (T)	ng/mL	8.0 ± 4.1	4.9 (1.3–28.2)	68%	Low	N/A	N/A	N/A	N/A
TGFB3 (B)	pg/mL	3133 ± 800	3500 (1600–4300)	44%	Low	N/A	N/A	N/A	N/A
TGFB3 (T)	ng/mL	119 ± 24.2	104 (67.6–181)	45%	Low	N/A	N/A	N/A	N/A
IFNG	pg/mL	206 ± 124	51.8 (0.7–3600)	330%	High	46.4 ± 6.0	49.1 (0.7–110)	69%	Low
TNFA	pg/mL	111 ± 76.6	5.5 (0.2–3480)	566%	High	5.3 ± 0.6	4.6 (0.2–15.6)	67%	Low
CSF2	pg/mL	234 ± 67.9	191 (1.5–1009)	105%	Moderate	202 ± 5.5	193 (187–230)	9%	Low
CSF3	pg/mL	87.1 ± 41	23.4 (10.4–616)	182%	High	33.8 ± 7.7	19.1 (10.4–100)	82%	Moderate
CCL2	pg/mL	2056 ± 471	1139 (737–6500)	82%	Moderate	N/A	N/A	N/A	N/A
CCL4	pg/mL	50.6 ± 5.6	50.9 (6.9–85.8)	37%	Low	N/A	N/A	N/A	N/A
CXCL8	pg/mL	1001 ± 194	600 (7.2–7670)	133%	Moderate	776 ± 107	575 (7.2–3000)	92%	Moderate

*Note*: Coefficient of variation (CV) was calculated before and after outlier (OL) removal by ROUT test (Q = 0.1%). Variance was categorised as low (CV < 75%), moderate (CV = 75%−150%), or high (CV > 150%). To calculate CV, a minimum of four studies for a given cytokine were required. N/A = not applicable as no outliers were identified.

**FIGURE 2:**
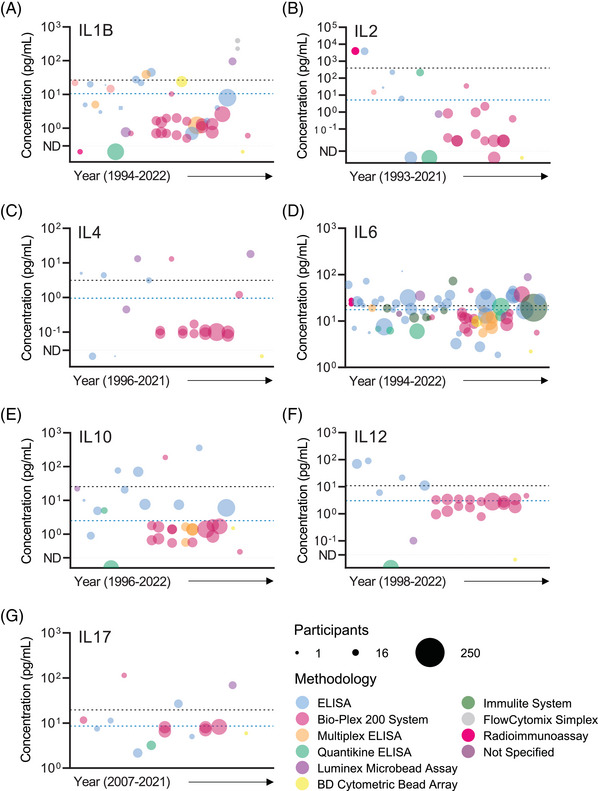
Bubble plot graphs visually representing studies investigating the abundance of IL1B (A), IL2 (B), IL4 (C), IL6 (D), IL10 (E), IL12 (F) and IL17 (G) in seminal plasma. Individual bubbles within the graph are representative of an individual study/cohort. Bubble size corresponds to number of participants in the study and bubble color indicates the method employed to quantify individual cytokines. Bubbles are ordered along the X‐axis from earliest (left) to most recent year (right). Bubbles presented below the horizontal dotted line and ND (not detectable, on Y‐axis) on individual graphs depict studies reporting the individual cytokine as being undetectable in human seminal plasma. Dashed lines: BLACK = mean concentration of individual cytokine with outliers included, and BLUE = mean concentration following removal of identified outlier(s).

Interleukin 1 beta (IL1B) is present at low to moderate levels in SP and is reported to be elevated in men with accessory sex gland infection,^39^ leukocytospermia,^14^, ^39^ varicocoele,^40^ chronic prostatitis,^25^ spinal cord injury,^41^ metabolic syndrome,^42^ HIV infection,^36^ and microbial infection^8^, ^10^, ^14^, ^15^ including COVID‐19 infection.^26^ Some studies reported higher IL1B in SP of infertile men,^43^, ^44^ but other studies showed no difference.^10^, ^15^, ^45^, ^46^, ^47^, ^48^, ^49^, ^50^, ^51^ IL1B concentration in SP of normozoospermic and/or fertile men was reported in 35 eligible studies published between 1994 and 2022,^8^, ^10^, ^14^, ^15^, ^25^, ^26^, ^36^, ^39^, ^40^, ^41^, ^42^, ^43^, ^44^, ^45^, ^46^, ^47^, ^48^, ^49^, ^50^, ^51^, ^52^, ^53^, ^54^, ^55^, ^56^, ^57^, ^58^, ^59^, ^60^, ^61^, ^62^, ^63^, ^64^, ^65^, ^66^ with a total of 1366 participants (Table 1), using mainly ELISA (18 of 35 studies) and multiplex microbead assays (14 of 35 studies). High variation in IL1B levels between studies was observed (CV = 280%), with a median (range) = 2.3 (0.5–391) pg/mL (Figure 2A, Table 2). Removal of outlier values reduced the variance (CV = 213%), with a median (range) = 1.7 (0.5–27.5) pg/mL (Table 2). There was a significant effect of assay platform on reported IL1B concentration (*p* < 0.001, Table 3). Pairwise comparison showed the FlowCytomix platform returned higher values than other platforms (*p* < 0.001), and the Luminex platform returned higher values than the Bio‐Plex 200 (*p* = 0.016) (Table 3).

**TABLE 3:** Analysis of the effect of assay platform on the reported concentrations of various cytokines

Cytokine	Assay Platform	ELISA	Bio‐Plex 200	Multiplex ELISA	Quantikine ELISA	Luminex Microbead	CBA	Immulite	FlowCytomix	RIA	Lateral Flow
IL1B	*p* < 0.001	13.7 ± 3.4^acd^	1.7 ± 0.5^c^	15.0 ± 12.0^acd^	14.7^acd^	47.8 ± 47.1^d^	12.1 ± 12.0^acd^		308 ± 82.5^b^	ND	
IL2	*NS*	685 ± 632	2.8 ± 2.4		107.5	0.76	ND			3962.7	
IL4	*p* = 0.026	2.5 ± 2.4^a^	1.2 ± 1.0^a^			10.5 ± 9.1^b^	ND				
IL6	NS	25.4 ± 3.6	14.6 ± 2.9	11.8 ± 5.3	10.4 ± 2.8	61.5 ± 26.5	6 ± 3.8	23.3 ± 8.3		25.7 ± 2.1	
IL7	NS		680 ± 138	557 ± 69.2							
IL10	NS	53.0 ± 31.4	14.4 ± 13.2	1.24 ± 0.2	2.5 ± 2.5		ND				
IL11	NS	3357 ± 1869			35.2 ± 16.7						
IL12	*p* = 0.007	39.6 ± 16.9^a^	2.6 ± 0.35^b^	2.3 ± 0.4^b^	ND	0.1	ND				
IL17	NS	10.5 ± 4.3	23.4 ± 15.1		3.2	69.0	5.9				
IL18	NS	160 ± 48.2	67	381	363 ± 92.5						
TGFB1 (T)	NS	194 ± 49.5	76.3		97.2 ± 4.8						
CSF2	NS	3.6 ± 2.1	275 ± 73.5								
CSF3	NS	144 ± 53.3	72.8 ± 49.5								
IFNG	NS	332 ± 297	132 ± 77.1			41.8 ± 40.2	ND				
TNFA	NS	141 ± 128	13.8 ± 8.5			45.3 ± 42.7	4.5 ± 2.3	4.2 ± 0.2		1057	
CCL2	*p* = 0.011	4640 ± 1860^a^	1586 ± 328^b^								
CXCL8	*p* = 0.001	1072 ± 231^ac^	338 ± 225^b^		2403 ± 581^c^	4485 ± 711^d^	1539 ± 711^abc^				664 ± 1006^abc^

*Note*: Linear mixed model analysis was performed for all cytokines to determine the effect of assay platform, fertility status, centrifugation, and storage of samples on variance in cytokine concentration. To undertake mixed model analysis, a minimum of five studies for a given cytokine were required, and a minimum of two studies assigned to the different assay platforms. There was insufficient data to reliably assign effects of fertility status, centrifugation, or storage of samples. When significant effects of assay platform were identified, pairwise comparisons between assay platforms were determined, and mean ± SEM for each assay platform are indicated. ND = Not detectable. Different superscript letters indicate significant difference between assay platforms (*p* < 0.05).

Soluble interleukin 1 receptor antagonist (sILRA) is reported to be increased in SP of men with leukocytospermia^22^ and HIV infection.^36^ Whether SP sIL1RA is altered in infertile men is unclear, with one study reporting no association,^22^ and another reporting increased sIL1RA in astheno‐, oligoastheno‐, and oligoasthenozoospermic men with genital infection.^45^ sIL1RA in SP of normozoospermic and/or fertile men was investigated in four studies published between 1996 and 2016,^22^, ^36^, ^45^, ^67^ with a total of 41 participants (Table 1). All studies had participant numbers of 15 or less, and most (three of four studies) utilised ELISA, except for one study using a microbead assay. Low variation in sIL1RA level between studies is observed (CV = 48%), with a median (range) = 204 (120–349) pg/mL (Table 2). Insufficient studies were available for analysis of effects of assay platform.

IL2 in SP is consistently elevated in individuals with evidence of genital infection.^16^, ^21^, ^23^, ^68^ Some studies report elevated IL2 in SP of infertile men compared to men of proven fertility,^23^, ^69^ while others report no difference.^23^, ^51^, ^70^ IL2 concentration in SP has been investigated in a total of 768 participants in 19 studies published between 1993 and 2021 (Table 1).^16^, ^21^, ^23^, ^34^, ^36^, ^37^, ^49^, ^51^, ^55^, ^56^, ^58^, ^59^, ^60^, ^66^, ^67^, ^68^, ^69^, ^70^, ^71^ Most studies used either ELISA‐based technology (eight of 19 studies) or multiplex microbead‐based assays (eight of 19 studies) for quantification. High variation in IL2 levels between studies was observed (CV = 294%), with a median (range) = 0.80 (0.02‐3963) pg/mL (Figure 2A, Table 2). Removal of outlier values reduced the variance (CV = 201%), with a median (range) = 0.4 (0.02–34.3) pg/mL (Table 2).

Soluble interleukin 2 receptor (sIL2R) may be elevated in SP of men with asthenozoospermia,^72^ but is not different in subfertile men.^5^, ^43^, ^71^, ^73^ sIL2R in SP was investigated in 192 men in eight included studies published between 1994 and 2016^5^, ^35^, ^36^, ^43^, ^67^, ^71^, ^72^, ^73^ (Table 1), with ELISA being the most common method of analysis (six of eight studies) (Figure S1C). Moderate variation in sIL2R levels between studies was observed (CV = 101%), with a median (range) = 1549 (21.2–5956) pg/mL (Figure S2C, Table 2). There were insufficient studies to analyse effects of assay platform.

IL4 in SP is reported to be inversely correlated with the presence of anti‐sperm antibodies^51^ and may be reduced in SP from men with bacterial infections^21^ but is not associated with fertility status.^51^, ^74^, ^75^ IL4 concentration in SP of a total of 635 normozoospermic and/or proven fertile men was investigated in 17 studies published between 1996 and 2021 (Table 1).^5^, ^21^, ^34^, ^36^, ^49^, ^51^, ^55^, ^58^, ^59^, ^60^, ^65^, ^66^, ^67^, ^68^, ^74^, ^75^, ^76^ There was a high level of variance between reported IL4 concentrations (CV = 174%), with a median (range) = 0.1 (0.08–18) pg/mL (Figure 2C, Table 2). There was a significant effect of assay platform (*p* = 0.026), and pairwise comparison showed the Luminex platform returned higher values than the Bio‐Plex 200 and ELISA assay platforms (*p* = 0.003 and *p* = 0.021, respectively) (Table 3).

IL5 is reported to be significantly increased in HIV‐positive men^36^ and decreased in men with oligo‐, astheno‐ and oligoasthenozoospermia.^76^ The abundance of IL5 in SP of 580 normozoospermic and/or proven fertile men was investigated in nine studies published between 2007 and 2020, with all utilising either Bio‐Plex 200 (eight of nine studies) or Luminex microbead (of 9 studies) methodologies (Figure S1D).^36^, ^37^, ^51^, ^55^, ^56^, ^58^, ^59^, ^60^, ^76^ A low level of variation was observed (CV = 36%), with median (range) = 33.2 (16.5–57.1) pg/mL (Table 2).

IL6 in SP has been studied extensively and shows strong potential as a biomarker for genital tract inflammation and/or infection, and disease. Many studies report elevated IL6 in SP from infertile men compared to fertile men^5^, ^6^, ^52^, ^77^, ^78^, ^79^, ^80^, ^81^, ^82^ and negative correlations between IL6 and sperm quality parameters.^5^, ^17^, ^52^, ^75^, ^82^, ^83^, ^84^ However, several others report no difference in sub‐fertile men^10^, ^17^, ^23^, ^43^, ^45^, ^46^, ^47^, ^50^, ^85^, ^86^, ^87^, ^88^, ^89^ and no relationship with sperm parameters.^22^, ^39^, ^45^, ^78^, ^85^ IL6 is also increased in SP samples from men with accessory gland infection^22^, ^39^, ^87^, ^88^ and/or genital infection and inflammation,^8^, ^10^, ^15^, ^23^, ^75^, ^82^ leukocytospermia,^14^, ^17^, ^19^, ^22^, ^39^, ^68^, ^79^, ^83^, ^90^ varicocoele,^15^, ^81^, ^83^, ^87^ prostatitis,^11^, ^24^, ^91^, ^92^, ^93^ idiopathic testicular lesions,^88^ recent COVID‐19 infection,^26^ metabolic syndrome/obesity,^42^, ^94^ and diabetes mellitus.^95^ IL6 in SP is positively associated with the abundance of leukocytes in semen.^17^, ^22^, ^24^, ^39^, ^75^, ^87^ SP IL6 was investigated in 64 studies published between 1994 and 2022 and comprising a total of 2966 normozoospermic and/or proven fertile men (Table 1).^5^, ^6^, ^8^, ^10^, ^11^, ^14^, ^15^, ^17^, ^19^, ^22^, ^23^, ^24^, ^26^, ^35^, ^36^, ^37^, ^39^, ^42^, ^43^, ^45^, ^46^, ^47^, ^49^, ^50^, ^51^, ^52^, ^53^, ^55^, ^56^, ^57^, ^58^, ^59^, ^60^, ^63^, ^64^, ^65^, ^67^, ^68^, ^77^, ^78^, ^79^, ^80^, ^81^, ^82^, ^83^, ^84^, ^85^, ^86^, ^87^, ^88^, ^89^, ^90^, ^91^, ^92^, ^93^, ^94^, ^95^, ^96^, ^97^, ^98^, ^99^, ^100^ Several methodologies have been utilised including ELISA (36 of 64 studies), multiplex microbead‐based assays (14 of 64 studies) and the Immulite 2000 automated chemiluminescence immunoassay analyser (seven of 64 studies) (Figure 2D). There was a moderate level of variance (CV = 93%) in reported concentrations of IL6, with median (range) = 15.9 (0.7–118) pg/mL. After removal of outliers the level of variance was low (CV = 67%), with median (range) = 15.0 (0.7‐60.2) pg/mL (Table 2). There was no detectable effect of assay platform (Table 3).

IL7 in SP appears not to be associated with fertility status,^37^, ^60^ reactive oxygen species,^55^, ^59^ or HIV infection.^36^ IL7 concentration in SP of normozoospermic and/or proven fertile men was investigated in 5 studies published between 2007 and 2020, with 304 participants (Table 1).^36^, ^37^, ^55^, ^59^, ^60^ All studies utilised either multiplex microbead‐based assays or multiplex ELISA assays (Figure S1E). The level of variance between studies was low (CV = 20%) in reported concentrations of IL7, and median (range) = 610 (507‐850) pg/mL (Table 2). There was no significant effect of assay platform on IL7 concentration (Table 3).

The abundance of IL9 in SP was investigated in just one eligible study published in 2016 and comprised of 10 individuals, with a reported median concentration of 367 pg/mL and no association with HIV infection^36^ (Figure S1F, Table 1).

IL10 is reported to be reduced in SP from infertile men with sperm defects,^78^, ^101^ and men with current genital tract infection^73^ or chronic bacterial prostatitis.^11^, ^93^ Other studies show IL10 is elevated in SP from men with sperm abnormalities,^86^ men with leukocytospermia,^102^ and men with recent COVID‐19 infection,^26^ while others find no association with sperm parameters or fertility status.^73^, ^76^, ^103^ IL10 abundance in SP of normozoospermic and/or proven fertile men was investigated in 1186 participants in 24 studies published between 1996 and 2022^11^, ^26^, ^36^, ^37^, ^49^, ^51^, ^55^, ^56^, ^59^, ^60^, ^63^, ^64^, ^66^, ^67^, ^73^, ^76^, ^78^, ^86^, ^93^, ^96^, ^101^, ^102^, ^103^, ^104^ (Table 1). Several different methodologies were utilised, with ELISA (10 of 24 studies) and multiplex microbead assay (11 of 24 studies) most common (Figure 2E). There was a high level of variance between studies (CV = 280%), with a median (range) = 1.6 (0.1–357) pg/mL. After removal of outliers, the level of variance was moderate (CV = 105%), with median (range) = 1.4 (0.1–10) pg/mL (Table 2). No effect of methodology on reported IL10 concentration was detected (Table 3).

Despite IL11 being reported to be significantly higher in SP samples from men with oligoasthenozoospermia and current infection,^23^ most studies reported no differences between fertile men and men with azoospermia, oligo‐, astheno‐, or oligoasthenozoospermia.^86^, ^105^ IL11 in SP of normozoospermic and/or proven fertile men was assessed in 110 participants in 5 studies published between 1996 and 2011 (Figure S1G, Table 1).^23^, ^67^, ^86^, ^96^, ^105^ Moderate variation in IL11 level between studies was observed (CV = 144%), with median (range) = 51.8 (9–6472) pg/mL (Table 2), with the three earliest studies reporting a mean of <60 pg/mL, and two others reporting median concentrations of 3575 and 6472 pg/mL,^86^, ^96^ despite all using ELISAs from the same manufacturer. There was no significant effect of assay platform on IL11 concentration (Table 3).

For IL12, many studies report no difference in SP concentration between fertile and sub‐fertile men^51^, ^73^, ^86^, ^96^, ^106^ or men with leukocytospermia.^107^ Other studies however report lower IL12 concentrations in infertile men^50^, ^107^ and infertile men with anti‐sperm antibodies,^106^ compared to fertile individuals. IL12 in SP of normozoospermic and/or proven fertile men was investigated in a total of 885 participants in 17 studies published between 1998 and 2022^37^, ^49^, ^50^, ^51^, ^55^, ^56^, ^57^, ^58^, ^59^, ^60^, ^63^, ^66^, ^67^, ^73^, ^86^, ^96^, ^106^, ^107^ (Table 1). ELISA technology from various manufacturers was used in six studies and multiplex microbead assays in 10 studies (Figure 2F). High variation in SP IL12 concentration was reported between studies (CV = 212%), with a median (range) = 3.0 (0.1–90) pg/mL. After removal of outliers variance was moderate (CV = 78%), with a median (range) = 2.8 (0.1–11) pg/mL (Table 2). A significant effect of assay platform on IL12 concentration was observed (*p* = 0.007), with pairwise comparison identifying differences between concentrations reported using ELISA assays and the Bio‐Plex 200 system and multiplex ELISA platforms (*p* = 0.010 and *p* = 0.040 respectively) (Table 3).

Several studies have investigated IL13 in SP, but none found a relationship with fertility status or semen quality.^59^, ^60^ IL13 in SP of normozoospermic and/or proven fertile men was assessed in 614 participants in eight studies published between 2007 and 2020^36^, ^37^, ^49^, ^55^, ^56^, ^59^, ^60^, ^76^ that all used the Bio‐Plex 200 system (Figure S2A). A high level of variation was observed between studies (CV = 307%), with median (range) = 0.4 (0.3–48.8) pg/mL. Following removal of outliers, the variance was reduced to low (CV = 21%), with median (range) = 0.4 (0.3–0.6) pg/mL (Table 2).

IL15 was investigated in only one eligible study, published in 2016, and comprising 10 participants, that utilised microbead analysis to define its relationship with HIV infection.^36^ The authors reported IL15 as being elevated in the SP of men with HIV, with a median concentration in normozoospermic and/or proven fertile men of 75 pg/mL (Figure S2B, Table 1).

IL16 in SP from proven fertile men was investigated in 21 participants in two studies, published in 2016 and 2022 (Table 1), with median concentrations of 10.7 and 144 pg/mL reported^36^, ^63^ (Table 1, Figure S2C). Both studies used the same microbead‐based assay platform for IL16 quantification. IL16 concentration in SP was not associated with either HIV infection^36^ or recurrent pregnancy loss.^63^

IL17 is reported to be elevated in SP of infertile men,^75^, ^101^, ^108^ although another study found no difference in men with various sperm abnormalities.^109^ IL17 is reported to be elevated in SP from men with genital tract inflammation,^75^ varicocoele,^109^ chronic prostatitis,^25^ Hepatitis B^108^ and diabetes mellitus^110^ compared to healthy normozoospermic men. IL17 concentration in SP of normozoospermic and/or proven fertile men was investigated in 469 participants in 13 studies published between 2007 and 2021^25^, ^36^, ^37^, ^59^, ^60^, ^64^, ^65^, ^66^, ^67^, ^75^, ^101^, ^108^, ^109^, ^110^ (Table 1). Most studies employed either ELISA‐based assays (six of 13 studies) or multiplex microbead assays (6 of 13 studies) for quantification (Figure 2G). IL17A is the most investigated of the five IL17 subunits and is usually referred to simply as IL17, so it is likely that this subunit was measured in most if not all these studies. There was high variation in IL17 level between studies (CV = 158%), with median (range) = 8 (2.2–114) pg/mL. After removal of outlier values, the degree of variance was low (CV = 71%), with median (range) = 7.5 (2.2–26.8) pg/mL (Table 2). There was no effect of assay platform on reported IL17 concentration (Table 3).

It is unclear whether IL18 in SP is associated with fertility status. One study reported there being no difference in IL18 concentration between normozoospermic and sub‐fertile men,^44^ while another reported SP IL18 as being elevated in infertile men,^108^ and a third described a negative correlation between SP IL18 and sperm concentration and motility.^7^ In addition, IL18 is reported as being higher in the SP of men with diabetes mellitus,^110^ genital tract infection,^7^ Hepatitis B infection,^108^ spinal cord injury^41^ and varicocoele,^111^ compared to normozoospermic and fertile healthy men. IL18 concentration was assessed in SP of 233 normozoospermic and/or proven fertile men in 9 studies published between 2006 and 2022^7^, ^36^, ^41^, ^44^, ^61^, ^63^, ^108^, ^110^, ^111^ (Table 1). Most studies utilised ELISA platforms (six of nine studies) or microbead assays (Figure s2d). A moderate level of variation in IL18 concentration was seen between studies (CV = 78%), median (range) = 170 (2.2–456) pg/mL (Table 2). There was no effect of assay platform on IL18 concentration (Table 3).

Some additional cytokines have been evaluated in only a small number of studies. IL23 concentration is reported to be increased in infertile men with abnormal sperm parameters compared to normozoospermic and fertile men.^75^, ^80^ IL23 was detected using ELISA technology in two studies, published in 2011 and 2014, and comprising 45 normozoospermic and fertile men (Table 1) at median and mean concentration of 5.9 and 2.2 pg/mL, respectively^75^, ^80^ (Figure S2E). IL33 was reported to be undetectable by ELISA in SP from 11 men in a single study published in 2021, including in men with varicocoele and genital infections^112^ (Figure S2F, Table 1). One study, published in 2017, reported SP IL37 concentration to be significantly higher in infertile men with varicocoele, compared to healthy fertile men.^111^ IL37 was detected using ELISA technology in 75 fertile men at a mean concentration of 109 pg/mL^111^ (Figure S2G, Table 1).

The concentration of leukemia inhibitory factor (LIF) in SP was found to be no different between healthy fertile men and men with HIV infection.^36^ Of the two studies to quantify LIF in SP, one (published in 2016) used microbead‐based technology while the other (published in 1996) used an ELISA, with mean LIF concentrations of 1.3 and 115 pg/mL respectively, being reported^36^, ^67^ (Figure S2H).

#### 3.4.2 Transforming growth factor beta superfamily members

In mammalian species, there are three TGFB isoforms, namely TGFB1, TGFB2 and TGFB3, all of which are present in varying abundance in human SP. All three isoforms of TGFB are produced in a latent, inactive form as disulphide‐linked dimers bound to a latency‐associated peptide, which is later cleaved to liberate active dimers capable of ligating to their cognate receptors and eliciting their biological function.^113^ This is also true of TGFB present in SP, where all three isoforms exist predominantly in a latent form before becoming activated once intromitted into the female reproductive tract at coitus.^114^, ^115^ This is an important consideration when measuring TGFB in SP. Endogenously active (bioactive) TGFB must be measured in untreated samples, while to measure the ‘total’ amount (bioactive + acid activated) of TGFB potentially available for release in SP, samples must be transiently acidified to release active TGFB from its precursor prior to assay.

**FIGURE 3:**
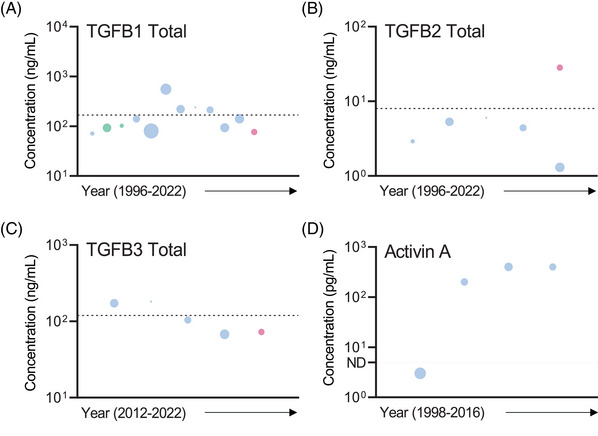
Bubble plot graphs visually representing studies investigating the abundance of total TGFB1 (A), total TGFB2 (B), total TGFB3 (C) and Activin A (D) in seminal plasma. A detailed legend describing bubble plot size and color as well as annotations is provided within the figure and figure legend text of Figure 2. Bubbles presented below the horizontal dotted line and ND (not detectable, on Y‐axis) on individual graphs depict studies reporting the individual cytokine as being undetectable in human seminal plasma. Dashed lines: BLACK = mean concentration of individual cytokine with outliers included, and BLUE = mean concentration following removal of identified outlier(s).

TGFB1 has been identified as a key cytokine involved in mediating the female immune response to seminal fluid.^53^, ^116^, ^117^ No difference in the concentration of endogenously active and total TGFB1 in SP has been reported between fertile and infertile men, or men with leukocytospermia.^47^, ^102^, ^118^ The abundance of TGFB1 in SP of normozoospermic and/or proven fertile men was investigated in 15 eligible studies published between 1996 and 2022 (Table 1), with 3 quantifying only endogenously active TGFB1,^26^, ^53^, ^119^ 4 quantifying only total TGFB1 (following acid activation)^63^, ^120^, ^121^, ^122^ and eight studies reporting both bioactive and total TGFB1 concentration.^12^, ^37^, ^47^, ^67^, ^102^, ^116^, ^117^, ^118^ Of the included studies, the most common method used for assessing TGFB1 concentration was ELISA (14 of 15 studies). The reported concentration of TGFB1 in SP is markedly higher following transient acidification, compared to untreated samples that quantified bioactive TGFB1, with latent TGFB1 being reported as accounting for greater than 90% of the total TGFB1 in the ejaculate. Bioactive TGFB1 has a moderate level of variation between studies (CV = 145%), with a median (range) = 1100 (200–9200) pg/mL (Figure S3A, Table 2). Total TGFB1 also had a moderate level of variation between studies (CV = 80%), with a median (range) = 121 (71–554) ng/mL (Figure 3A, Table 2). This may be because of differences in the methodology employed to achieve acid activation of the SP sample.

TGFB2 contributes to the immune‐regulatory effects of SP in female reproductive tract cells,^116^, ^117^ with some evidence that SP of fertile normozoospermic men contains less TGFB2 compared to SP of men with reduced semen quality.^121^ TGFB2 abundance was quantified in SP of normozoospermic and/or proven fertile men in seven studies published between 1996 and 2022, with one study investigating TGFB2 only in its bioactive form,^53^ two studies investigating only total TGFB2 after acid activation^63^, ^121^ and four studies investigating both active and total TGFB2^12^, ^67^, ^116^, ^117^ (Table 1). All included studies had 25 or fewer participants, with all but one study employing ELISA platforms to quantify TGFB2. Low between‐study variation was observed in the reported concentrations of endogenously active and total TGFB2 across the studies (Figure 3B and Figure S3B). A moderate level of variation in bioactive TGFB2 concentration (CV = 125%) was reported, with a median (range) = 220 (8–300) pg/mL, while a low level of variation was observed for total TGFB2 (CV = 68%), with median (range) = 4.9 (1.3–28.2) ng/mL (Table 2).

TGFB3 also contributes to the immune‐regulatory effects of SP in female reproductive tract cells.^116^, ^117^ TGFB3 concentration is reported to be lower in SP from men with reduced semen quality,^121^ although there is high between‐individual variation.^12^ TGFB3 abundance in SP of normozoospermic and/or proven fertile men was investigated in five studies published between 2012 and 2022, with 2 quantifying the endogenously active form,^63^, ^121^ and three studies quantifying both the active and latent forms^12^, ^116^, ^117^ (Table 1). All studies except for one utilised ELISA‐based assays (bioactive TGFB3: Figure S3C; Total TGFB3: Figure 3C). Similar to TGFB1 and TGFB2, TGFB3 concentration was substantially greater following acid activation, with a low level of variation between studies in the reported concentration of both bioactive and total TGFB3 (CV = 44% and 45% respectively), and median (range) = 3500 (1600–4300) pg/mL and 104 (67.6–181) ng/mL, respectively (Table 2).

Activin A, inhibin B, follistatin (FST), and growth differentiation factor 15 (GDF‐15) are all members of the TGFB superfamily, with established roles in immune regulation. Current understanding of their roles in SP is limited. The concentration of these factors was investigated in only a small number of eligible studies (four studies published between 1998 and 2016, one study published in 1998, two studies published between 2015 and 2016, and one study published in 2010, respectively [Table 1]) in SP of normozoospermic and/or proven fertile men, and each of these studies had low participant numbers. There was a high degree of conformity across the studies, potentially because of use of similar ELISA platforms. The mean concentration of activin A reported in the included studies ranged from undetectable up to a mean concentration of 399 pg/mL, mean inhibin B concentration was 1.4 ng/mL, while mean and median FST concentration was 46 and 70 ng/mL respectively, and median GDF‐15 concentration was 1.8 μg/mL (Figure 3D and Figures S3D–F, respectively).

#### 3.4.3 Interferons

Understanding of interferon alpha (IFNA) actions in male reproduction is limited; however, a role in protecting the testes against viral infection seems likely. IFNA is reported to be more abundant in SP of men with recent COVID‐19 infection compared to those without.^26^ Furthermore, IFNA is reported to be substantially higher in men with oligozoospermia compared to normozoospermic men, suggesting a role in spermatogenesis.^123^ Three studies published between 1998 and 2021 and comprising 146 normozoospermic and/or proven fertile men were included in the current analysis^26^, ^36^, ^123^ (Table 1) with a large amount of between‐study variation observed in reported IFNA concentrations (Table 2). Two studies (one using an ELISA, one with platform unspecified) reported mean IFNA concentrations in SP of 92.0 and 90.0 ng/mL,^36^, ^123^ while a third study using a microbead‐based assay reported a median concentration of just 243 pg/mL^36^ (Figure S4A).

**FIGURE 4:**
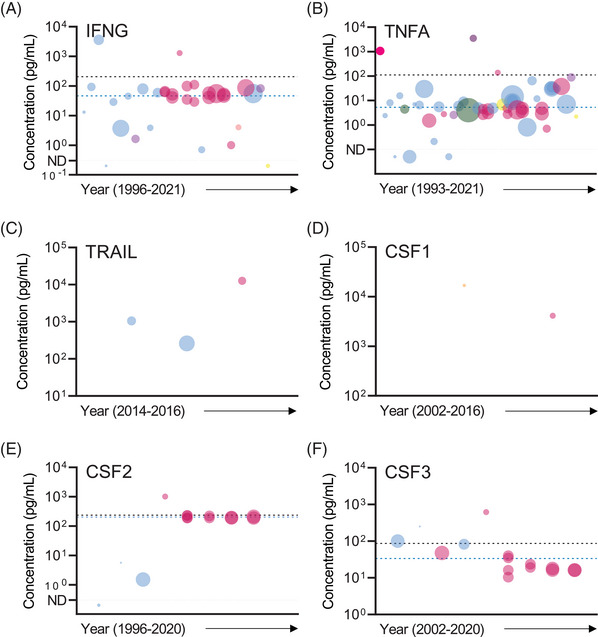
Bubble plot graphs visually representing studies investigating the abundance of IFNG (A), TNFA (B), TRAIL (C), CSF1 (D), CSF2 (E) and CSF3 (F) in seminal plasma. A detailed legend describing bubble plot size and color as well as annotations is provided within the figure and figure legend text of Figure 2. Bubbles presented below the horizontal dotted line and ND (not detectable, on Y‐axis) on individual graphs depict studies reporting the individual cytokine as being undetectable in human seminal plasma. Dashed lines: BLACK = mean concentration of individual cytokine with outliers included, and BLUE = mean concentration following removal of identified outlier(s).

Interferon gamma (IFNG) is a potent immunoregulatory cytokine with established roles in both innate and adaptive immunity. IFNG abundance in SP is highly variable between different studies, and fluctuates within individuals over time.^13^, ^51^ IFNG is usually undetectable or present in low concentrations in the SP of fertile men but can be substantially elevated in men with obstructive azoospermia^86^ or leukocyospermia,^68^ chronic prostatitis,^25^ inflammatory disease^75^ or recent COVID‐19 infection.^26^ It has been reported that IFNG abundance in SP is unchanged in men with HIV^36^ or in the presence of genital tract infection.^7^Some studies report negative correlations between SP IFNG abundance and indicators of sperm quality, including total count, morphology and motility,^51^, ^124^ but others contradict this.^52^, ^123^ There is evidence to suggest IFNG can suppress the female immune response induced by SP,^125^ and this may be clinically important as SP IFNG content is associated with idiopathic infertility in some couples.^62^ A total of 26 studies, published between 1996 and 2021, have investigated IFNG in SP of 1120 normozoospermic and/or proven fertile men^7^, ^13^, ^25^, ^26^, ^34^, ^36^, ^37^, ^49^, ^51^, ^55^, ^56^, ^58^, ^59^, ^60^, ^64^, ^65^, ^66^, ^67^, ^68^, ^75^, ^76^, ^86^, ^96^, ^123^, ^124^ (Table 1). Several different assay platforms and assay manufacturers were utilised across the included studies, the most common before 2015 being ELISA (13 of 26 studies), with a shift toward microbead‐based platforms (11 of 26 studies) in recent years (Figure 4A). There was a high level of variance in the reported concentrations of IFNG between studies (CV = 330%), with median (range) = 51.8 (0.7–3600) pg/mL. After removal of outliers the level of variance was reduced was low (CV = 69%) with median (range) = 49.1 (0.7–110) pg/mL (Table 2). There was no effect of assay platform on reported IFNG concentration (Table 3).

#### 3.4.4 TNFs

Tumor necrosis factor (TNFA, also known as TNF) is a pro‐inflammatory cytokine with established roles in immune regulation and inflammation. It is produced predominantly by macrophages and other immune cell subsets in response to infection and other pro‐inflammatory stimuli. TNFA is reported to be elevated in SP of men with obstructive azoospermia,^86^, ^101^ and a negative correlation between TNFA concentration and sperm motility has been reported,^51^, ^85^, ^126^ although many other studies report no difference in SP TNFA concentration between fertile men and subfertile men.^8^, ^10^, ^14^, ^15^, ^45^, ^47^, ^48^, ^50^, ^59^, ^70^, ^77^, ^80^, ^81^ Multiple studies have shown elevated TNFA in SP of men with genitourinary infection or disease, including men with leukocytospermia,^68^, ^83^, ^90^, ^127^ microbial infection,^10^ varicocoele,^15^, ^83^ chronic bacterial prostatitis,^11^, ^93^ HIV infection^36^ and recent COVID‐19 infection,^26^ implying SP TNFA may indicate underlying inflammation, infection, and genitourinary disease in men. TNFA in SP of 2142 normozoospermic and/or proven fertile men was investigated in 41 studies published between 1993 and 2021^8^, ^10^, ^11^, ^14^, ^15^, ^26^, ^34^, ^36^, ^37^, ^42^, ^45^, ^47^, ^48^, ^49^, ^50^, ^51^, ^55^, ^56^, ^59^, ^60^, ^64^, ^65^, ^66^, ^67^, ^68^, ^70^, ^77^, ^80^, ^81^, ^83^, ^85^, ^86^, ^90^, ^93^, ^94^, ^95^, ^96^, ^98^, ^101^, ^126^, ^127^ (Table 1). There was considerable variation in the mean concentrations of SP TNFA, ranging from undetectable to 3480 pg/mL, although <50 pg/mL was most consistently reported. Six different assay platforms from various manufacturers were used to quantify TNFA, with the most common being ELISA (21 of 41 studies) and, from 2007 onward, microbead‐based assays (13 of 41 studies) (Figure 4B). There was a high level of variance in the reported concentrations of TNFA between studies (CV = 566%), with median (range) = 5.5 (0.2–3480) pg/mL. After removal of outliers, variance was reduced to a low level (CV = 67%), with median (range) = 4.6 (0.2–15.6) pg/mL (Table 2). There was no effect of assay platform on TNFA concentration (Table 3).

TNFB and TNF‐related apoptosis‐inducing ligand (TRAIL) in SP have not been extensively studied. Elevated TRAIL in SP of infertile men with varicocoele compared to SP of fertile men has been reported,^128^ as well as in men exhibiting chronic inflammation of the genitourinary tract,^75^ but not in men infected with HIV.^36^ Only two studies (published in 1996 and 2016) investigated TNFB in SP of normozoospermic and/or proven fertile men, while three (published in 2014 and 2016) investigated TRAIL. These studies had low participant numbers, with TNFB and TRAIL measured in 13 and 60 men, respectively (Table 1). Large differences were observed in the reported concentration of TNFB between the two studies, with median values below the detectable limit in one study but 54.5 pg/mL in the other^36^, ^67^ (Table 2). Large variation was also observed for TRAIL, with a mean of 260 pg/mL in one study and a median of 12616 pg/mL in the other. Both ELISA and multiplex microbead assays were utilised to quantify TNFB and TRAIL (Figure S4B and Figure 4C, respectively).

#### 3.4.5 CSFs

CSFs are a small family of signaling proteins with important roles in immune modulation and tolerance. The abundance of CSF1, CSF2, and CSF3 in human SP was investigated in a total of 12 studies, with two studies investigating all three CSFs.

CSF1 (also known as macrophage CSF, M‐CSF) in SP appears unrelated to sperm parameters and fertility status.^34^ Two studies, published in 2002 and 2016, investigated CSF1 in SP of 63 normozoospermic and/or proven fertile men^34^, ^36^ (Table 1). The reported concentrations of CSF1 were substantially different between the two studies (median concentrations of 16800^34^ and 4110 pg/mL^36^), likely because of low sample size and use of different analytical platforms, with the earlier study utilising ELISA and the latter a multiplex microbead assay (Figure 4D).

CSF2 (also known as granulocyte‐macrophage CSF, GM‐CSF) in SP is not associated with semen quality,^59^, ^60^ with oxidative stress or metal exposure,^58^, ^60^, ^104^ or with HIV infection.^36^ Eight studies, published between 1996 and 2020, investigated CSF2 concentration in the SP of normozoospermic and/or fertile men^36^, ^37^, ^53^, ^58^, ^59^, ^60^, ^67^, ^104^ (Table 1). Earlier studies utilised ELISA before a shift to microbead‐based assays from 2007 onward (Figure 4E, Supporting Dataset 1). There was a moderate level of variation in the reported level of CSF2 concentration (CV = 105%), with median (range) = 191 (1.5–1009) pg/mL. After removal of outlier the level of variation was low (CV = 9%) with a median (range) = 193 (187–230) pg/mL (Table 2). There was no effect of assay platform on reported CSF2 concentration (Table 3).

CSF3 (also known as granulocyte G‐CSF) is reported to be increased in the SP of men with leukocytospermia^34^ but does not differ between men with and without sperm abnormalities,^34^, ^47^ or HIV infection.^36^ CSF3 was investigated in nine included studies, published between 2002 and 2020, and reporting on a total of 563 normozoospermic and/or proven fertile men^34^, ^36^, ^37^, ^47^, ^53^, ^58^, ^59^, ^60^, ^104^ (Table 1). The highest abundance of CSF3 was detected using ELISA, and lower levels are reported in recent studies using microbead‐based assays (Figure 4F). More than half of the studies (six of nine studies) reported CSF3 concentration to be undetectable or <85 pg/mL. There was a high level of variation between studies (CV = 182%), with median (range) = 23.4 (10.4–616) pg/mL. After removing outliers, variation was moderate (CV = 82%), with median (range) = 19.1 (10.4–100) pg/mL (Table 2). There was no effect of assay platform on CSF3 concentration (Table 3).

#### 3.4.6 Chemokines

Chemokines are potent chemoattractant cytokines, with important roles in the immune system where they selectively attract, recruit, and activate immune cells. The role of chemokines in SP is unclear, but it is likely these factors influence the female immune response through effects on both innate and adaptive immune cells. Chemokines are separated into four different families depending on the position of the cysteine residues (CXC, CX3C, CC and C), with the CXC and CC chemokines comprising the largest families.

#### 3.4.7 CC chemokine ligand family

The CC chemokine ligand (CCL) family is comprised of 28 family members, with seven family members investigated by studies included in this review. CCLs are known to induce cell migration through the binding of CC chemokine receptors and are involved in the chemotaxis of monocytes, DCs, natural killer cells, T cells, B cells, eosinophils and basophils.

**FIGURE 5:**
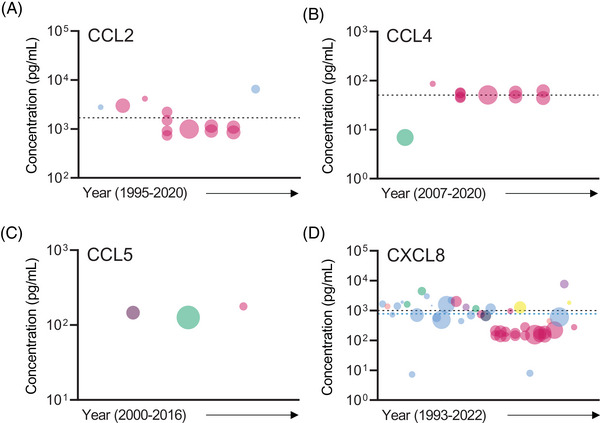
Bubble plot graphs visually representing studies investigating the abundance of CCL2 (A), CCL4 (B), CCL5 (C) and CXCL8 (D) in seminal plasma. The bubbles are ordered along the X‐axis from earliest (left) to most recent year (right). A detailed legend describing bubble plot size and color as well as annotations is provided within the figure and figure legend text of Figure 2. Bubbles presented below the horizontal dotted line and ND (not detectable, on Y‐axis) on individual graphs depict studies reporting the individual cytokine as being undetectable in human seminal plasma. Dashed lines: BLACK = mean concentration of individual cytokine with outliers included, and BLUE = mean concentration following removal of identified outlier(s).

CCL2 (also known as monocyte chemoattractant protein‐1/MCP‐1) is reported to be higher in SP of men with astheno‐ and oligoasthenozoospermia, compared to normozoospermic males^50^; however this is not a consistent finding.^19^, ^60^ CCL2 may be elevated in SP of leukocytospermic men^19^ but was not changed in men with HIV infection compared to uninfected men.^36^ Eight studies published between 1995 and 2020 have investigated CCL2 in SP of 548 normozoospermic and/or proven fertile men^19^, ^36^, ^37^, ^50^, ^59^, ^60^, ^98^, ^104^ (Table 1). All studies reported CCL2 to be highly abundant (Figure 5A, Supporting Dataset 1). Six studies utilised a microbead based assay and Bio‐Plex 200 system to quantify CCL2, and two studies used ELISA technology (Supporting Dataset 1). The degree of variation between studies was moderate (CV = 82%), with a median (range) = 1139 (737–6500) pg/mL (Table 2). Assay platform was identified as a determinant of variability in CCL2 levels (*p* = 0.011), with higher levels detected by ELISA assay compared to the Bio‐Plex 200 platform (*p* = 0.011) (Table 3).

CCL3 (also known as macrophage inflammatory protein 1 alpha/MIP‐1A) appears unchanged in men with HIV infection.^36^ CCL3 has been investigated in SP of 95 normozoospermic and/or proven fertile men in three eligible studies, published between 1996 and 2016, using ELISA and microbead platforms^36^, ^37^, ^67^ (Table 1). Low variation was observed in SP CCL3 concentration, with all three studies reporting values below 40 pg/mL despite using different assay platforms (Figure S5A).

CCL4 (also known as macrophage inflammatory protein 1B) has been in investigated in proven fertile men^37^ and in men with sperm abnormalities.^50^, ^59^, ^60^ One study reported higher abundance of CCL4 in men with oligo‐, astheno‐ and oligoasthenozoospermia,^50^ yet two other studies did not detect differences.^59^, ^60^ Another study found no relationship between metal exposures and CCL4 in SP.^104^ A total of six studies published between 2007 and 2020 examined CCL4 in SP of normozoospermic and/or proven fertile men, with a combined total of 539 participants^36^, ^37^, ^50^, ^59^, ^60^, ^104^ (Table 1). Of these, all but one utilised the Bio‐Plex 200 system to quantify CCL4 (Figure 5B). There was a low degree of variation between studies in reported CCL4 concentration (CV = 37%), with a median (range) = 50.9 (6.9–85.8) pg/mL (Table 2). There were insufficient studies to analyse effect of assay platform.

CCL5 (also known as regulated on activation, normal T cell expressed and secreted, RANTES) is significantly increased in the SP of individuals with HIV infection, compared to uninfected men^36^ but reduced in SP from infertile men with high levels of anti‐sperm antibodies.^129^ CCL5 was investigated in three studies, published between 2000 and 2016, with a total of 121 participants using ELISA and microbead platforms^36^, ^37^, ^129^ (Table 1). Despite the different methodologies, little variation was observed, with median values of 126, 146 and 177 pg/mL from the three studies (Figure 5C).

CCL7 (also known as macrophage chemotactic protein 3, MCP‐3), CCL11 (also known as eotaxin) and CCL27 (also known as cutaneous T cell–attracting chemokine, CTACK) in SP were all assessed in one small study published in 2016 and found to be present at low or moderate levels and unchanged by HIV infection status.^36^ CCL11 was also studied in a second small study published in 2010 comprising 16 normozoospermic men.^74^ Median values in SP of normozoospermic and/or proven fertile men were 114 pg/mL, 9.7‐75.4 pg/mL and 218 pg/mL, for CCL7, CCL11 and CCL27 respectively (Figure S5B–D, respectively, Table 1).

#### 3.4.8 Chemokine (C‐X‐C motif) ligand family

The C‐X‐C chemokine ligand family is comprised of 17 members, and to date, eight of these have been shown to be present in SP including CXCL1, CXCL5, CXCL6, CXCL8, CXCL9, CXCL10, CXCL11 and CXCL12. Other than CXCL8, the role(s) played by these chemokines in SP remain largely unknown.

CXCL1 (growth‐regulated alpha protein/GRO alpha) is reported to be higher in SP of men with leukocytospermia, compared to normal fertile men as well as infertile men without leukocytospermia^103^ but was unchanged in men with HIV.^36^ CXCL1 was investigated in 21 normozoospermic and/or proven fertile men in two included studies, published in 1995 and 2016, using ELISA and microbead assay platforms^36^, ^103^ (Table 1). One study reported a median CXCL1 concentration of 2840 pg/mL, and the other reported a median concentration of 2669 pg/mL (Figure S6A).

CXCL5 and CXCL6 were each investigated in one study, published in 2017 and 2008,^40^, ^130^ and with participant numbers of 60 and 14 respectively. The mean concentration of CXCL5 in fertile men was reported to be 248 pg/mL, with higher concentrations in the SP of men with varicocoele‐induced infertility.^40^ The sole study investigating CXCL6 in SP of fertile men reported a mean (range) concentration of 19 (5–47) nM (converted concentration: 5480 [1400–13,600] pg/mL) (Figure S6B,C).

CXCL8 (also known as IL8) has been extensively investigated for its association with male reproductive tract infection and leukocytospermia. A number of studies report that CXCL8 in SP is higher in men with reduced sperm quality, including those with asthenospermia,^23^, ^86^ and oligoasthenospermia^50^, ^80^ but not all studies report data consistent with this finding.^20^, ^22^, ^59^, ^60^, ^131^ There is evidence that CXCL8 is linked with male reproductive infection,^8^, ^13^, ^14^, ^15^, ^16^, ^17^, ^18^, ^19^, ^20^, ^21^, ^22^, ^23^ including men with recent COVID‐19 infection^26^ and men with chronic prostatitis.^24^, ^25^ The abundance of CXCL8 in SP was investigated in a total of 1501 normozoospermic and/or proven fertile men in 40 studies published between 1993 and 2022^8^, ^13^, ^14^, ^15^, ^16^, ^17^, ^18^, ^19^, ^20^, ^21^, ^22^, ^23^, ^24^, ^25^, ^26^, ^34^, ^36^, ^37^, ^42^, ^47^, ^49^, ^51^, ^53^, ^55^, ^56^, ^57^, ^58^, ^59^, ^60^, ^63^, ^64^, ^65^, ^66^, ^67^, ^80^, ^81^, ^86^, ^96^, ^131^ (Table 1). Five different methodologies were employed to measure CXCL8 concentration in SP, from diverse manufacturers. The most common platform was ELISA (23 of 40 studies), before a shift toward microbead‐based technology (14 of 40 studies) from 2009 onward (Figure 5D). There was a moderate level of variation between studies in the reported concentrations of CXCL8 (CV = 133%), with median (range) = 600 (7.2‐7670) pg/mL. After outlier removal the level of variation was still moderate (CV = 92%), with median (range) = 575 (7.2–3000) pg/mL (Table 2). There was a significant effect of assay platform on CXCL8 concentrations (*p* = 0.001), with pairwise comparisons indicating significant differences in values returned by several of the different platforms, and notable Luminex microbeads returning higher levels than all other platforms (*p* < 0.020) and a trending difference with the Quantikine ELISA platform (*p* < 0.054) (Table 3).

The concentrations of CXCL9, CXCL10 and CXCL11 in SP were each measured by ELISA in a single study of 14 participants, published in 2008.^132^ CXCL9 was the most abundant of the three with a mean (range) concentration of 25 (8.1–40.6) nM (converted concentration: 7210.5 [2336.2–11709.9] pg/mL), while CXCL10 was present at 1.8 (0.3–5.8) nM (converted concentration: 519.2 [86.5–1672.8] pg/mL) and CXCL11 at 0.6 (0.2–1.6) nM (converted concentration: 173.1 [57.7–461.5] pg/mL) (Figure S6D–F, respectively). Further, the authors reported CXCL9 to be reduced in men with history of vasectomy noting this was not the case for CXCL10 or CXCL11.

CXCL12 was investigated in two studies published in 2007 and 2016,^36^, ^37^ in a total of 69 normozoospermic and/or proven fertile men. There was a substantial difference in the median concentration of 803 pg/mL reported using ELISA in one study,^36^ compared to 5742 pg/mL using microbead‐based analysis in the other^37^ (Figure S6G).

## 4 DISCUSSION

### 4.1 Summary of evidence

Human SP contains a diverse array of cytokines that have known immune‐regulatory functions and potential to influence fertility and reproductive success in a range of ways. We extracted data from 118 published research articles that utilised various immunoassay techniques to quantify cytokines in SP of healthy normozoospermic and proven fertile men. Altered levels of many of these factors have been associated with reduced sperm quality, fertility status and/or various exposures or conditions of the male urogenital tract (summarised in Table S2 and S3). This points to the likely clinical utility of measuring SP cytokines as indicators of reproductive health and fertility status. However, the potential of SP cytokines as diagnostic markers cannot be realised until normal ranges for their concentrations in healthy men are defined.

### 4.2 Major findings

In the 118 eligible studies included in this review, a total of 51 individual cytokines were reported as being detectable in SP of healthy men. There is large variation between studies in the values obtained for individual cytokine levels. This variation is higher for some cytokines than others, including several that have strong potential for clinical utility. Cytokines IL1B, IL2, IL10, IL13, TNFA and IFNG showed the highest degree of between‐study variation. The low number of studies examining these factors is likely to a contributing factor to their high variation but was not the sole factor. Even factors such as IL6, TNF and CXCL8 that have each been measured in 20 or more studies showed more than a 100‐fold difference in the mean or median values from the highest to lowest reported data sets. Variation between studies may in part reflect population factors that contribute to biological variation in cytokine synthesis and differ between study cohorts. Nevertheless, the high degree of variance seen is more than can reasonably be accounted for by age, racial, lifestyle and socio‐economic factors, or differences in study participant numbers, or inclusion and exclusion criteria.

Mixed model analysis showed that technical factors are a driver of the high variation seen in several SP cytokines. In particular, the assay platform technology was shown to be a major contributing factor. Other technical factors including insufficient assay validation, and variation in protocols for sample collection, processing, and storage, are also likely to contribute. Each of these technical factors are considered in more detail below.

### 4.3 Technical factors contributing to variation in cytokine measurement in SP

Appropriate validation of cytokine assay platforms is a challenge for biological fluids such as SP that constitute complex matrices for immunoassays. ELISA, microbead assays and older RIA all involve immunoglobulin‐ligand binding, which is highly responsive to the biochemical features of the biological fluid being analysed, in ways that can impair or promote antibody‐ligand interactions, causing readings to be artificially elevated or reduced. With the lack of standardised approaches to measuring cytokines and chemokines in SP and the diverse range of assay platforms available to investigators, it is unsurprising that the reported concentrations for specific analytes vary greatly between studies. There are many technical factors that have potential to contribute to this variation. They can be broadly classified into two main categories: sample processing and storage, and assay selection and validation.

The first consideration is collection protocol and sample preparation. The WHO VI guidelines recommend that all semen samples are collected according to a standardized protocol including ensuring men adhere to the requisite 2–7 days of ejaculatory abstinence prior to producing a sample.^1^ An important consideration is ensuring ejaculate volume is precisely measured, thereby enabling researchers to report confidently on both the concentration and total ejaculate content of their analyte of interest. The WHO VI guidelines recommend SP should be prepared from whole semen by vigorous centrifugation (10–15 min at 3000 x g) to remove as much particulate matter as possible and stored at −20°C until analysis. This is important in cytokine analysis as the presence of any residual spermatozoa or cellular debris can cause interference and severely affect assay performance. Sample storage is also a source of variation between studies. In our experience (Sharkey et al., unpublished data), cytokine recovery is maximised when SP is stored in multiple small aliquots at −80°C, and repeated freeze thaw cycles are avoided, as this causes loss of protein structural integrity. Many cytokines are highly labile and storage of samples under suboptimal conditions, for example at −20°C for extended periods, repeated freezing and thawing, or holding at room temperature or 4°C for more than a short period prior to assay, can result in endogenous proteolytic activity and cause cytokine proteins to denature, such that reduced amounts will be detected in immunoassays. Addition of protease inhibitors may inhibit loss to some degree.

The quality and nature of the assay platform employed, together with their inherent differences in assay sensitivity and specificity, is the second major challenge. Most assays utilised in the 118 publications included in this analysis were ELISA, microbead, or (in some older studies) RIA assays. For each of these immunoassay systems, several criteria must be met to ensure that the results are accurate, reliable and reproducible. Many commercial assays may have been validated by the supplier or ‘in‐house’ for the quantification of specific proteins in serum, plasma, urine, saliva or cell culture supernatants. Nevertheless, it cannot be assumed they will perform comparably when used to detect cytokines in SP, a viscous biological sample with complex matrices. Whenever assay platforms are first applied to SP, appropriate assay evaluation and validation must first be performed. In broad terms, matrix effects would be expected to be stronger when SP is less diluted, and so would pose a greater problem in assays for cytokines present in low levels (necessitating less diluted samples), and for assays where detection thresholds are higher. From this perspective, microbead‐based assays offer benefits as they generally have lower detection limits (enabling higher dilution of sample), as well as requiring lower sample volume.

Characteristics such as specificity, sensitivity and cross‐reactivity are important considerations for assay selection, and it is important to understand the limitations of the chosen assay platform. For example, immunoassays are susceptible to interference from both exogenous and endogenous factors,^133^ resulting in the inaccurate reporting of the concentration of a specific protein's concentration in a specific biological fluid. Interference of this nature (often referred to as a ‘matrix effect’) is most relevant when immunoassays are used to measure compounds in viscous or complex biological fluids, such as SP. The factors that contribute to immunoassays reporting incorrect results, and the steps required to validate an assay for accurate quantification in a specific biological fluid, have been comprehensively described elsewhere (reviewed by Ghazal et al. 2022).^134^

### 4.4 Strengths and limitations

A strength of this study is the design and execution of the systematic literature review, which followed strict search strategies and enforced defined eligibility criteria, affording reproducibility in the results. The synthesis of information from a wide range of published studies provides a foundation from which a consensus can be reached on appropriate assay platforms and optimal validation strategies for SP analysis. Once this is achieved, studies to determine normal ranges for healthy men can progress.

We acknowledge several important limitations of this review. Firstly, most studies included relatively small groups of healthy normozoospermic and/or proven fertile men for comparison to various other groups. Healthy men are generally more difficult to recruit into clinical studies, compared to men with infertility or other disease states. The definition of normozoospermic has also changed across the six editions of WHO guidelines, with changes to the methodologies used to perform semen analysis and the resultant reference values likely also contributing, to some extent, to the high level of variation observed in reported cytokine concentrations. Furthermore, several studies omitted important information such as racial characteristics and exclusion criteria were not clearly stated or may not have been applied, potentially contributing to higher levels of variation within and between different studies. Secondly, we did not distinguish between normozoospermic and proven fertile men, and this may have increased heterogeneity in the characteristics of cohort participants. However, assessment by linear mixed model analysis indicated there was no significant effect of fertility status—that is, classification as normozoospermic or proven fertile—on variation in cytokine data (data not shown). A third potential limitation arises because of differences in the extent to which different molecules in SP have been measured. For example, IL6, TNF and CXCL8 were measured in 20 or more of the included studies while other factors were measured in just one or two studies, reducing confidence in the generalizability of the reported data. A final limitation is associated with differences in the way the data are presented. Limited participant information and differences in the methodologies used for quantification are a constraint on attempts to pool data and draw inferences on normal ranges based on these existing data sets.

### 4.5 Research recommendations and call to action

There is increasing evidence to suggest that normal sperm parameters alone are poor predictors of male fertility.^135^ Analysis of soluble factors in SP, in particular cytokines that are known to have physiological relevance for sperm health and/or the female immune response, holds promise as an approach to expand analyses of male fertility and reproductive health. However, before any clinical utility can be realised, appropriately powered studies are required to identify the cytokine factors of clinical value, and to define reference ranges for these factors that can be applied with confidence to different laboratories internationally, and act as ‘decision limits’ for assigning samples to clinically meaningful categories.

Protocols for the accurate evaluation of semen quality in men is standardised across clinics, through development and adherence to ISO Standard 23,162 for the basic examination of semen,^136^ on which the WHO VI laboratory manual for the examination and processing of human semen was based.^1^ There is no equivalent guide describing appropriate methods for the processing and storage of SP for cytokine and chemokine analysis.

Therefore, to achieve the goal of setting reference ranges or decision limits, research scientists and clinicians with experience in the field must first establish a set of agreed guidelines for accurately measuring cytokines and chemokines in human SP samples. In our opinion, these guidelines will need to incorporate agreed definitions on what participant cohort information should be reported, as well as provide a clear description of optimal methodology for the preparation and storage of SP for cytokine and chemokine analysis. Furthermore, the guidelines should make clear recommendations on the most appropriate platform to use for measuring cytokines and chemokines in SP and then describe the necessary steps required to validate assays to exclude matrix effects to ensure accurate, reliable and reproducible results are obtained.

Recently, an opinion piece from the European Society of Human Reproduction and Embryology Special Interest Group Andrology (SIGA) highlighted that even when standardized protocols and training for semen analysis are available, these are too frequently ignored, and studies that fail to follow the proven high‐quality methods in WHO VI continue to be published.^137^, ^138^ SIGA proposed authors in the fields of Andrology and Reproductive Medicine be instructed to stringently follow the laboratory methods detailed in WHO VI when examining human semen and include a completed checklist (adapted from Björndahl et al. 2015)^139^ when submitting their manuscript, so the quality of the semen evaluation method used can be readily assessed. We propose that measuring cytokines and chemokines in SP, as part of an extended semen analysis for fertility investigation in men, will require an equivalent ISO Standard (and checklist) to be developed.

## 5 CONCLUSION

Seminal plasma cytokines may be an informative parameter in diagnosing male infertility and sub‐fertility, but large between‐laboratory variation in reported concentrations is a major barrier to this goal. To expand understanding of the clinical utility of SP cytokines requires development of diagnostic standards, particularly the definition of normal reference ranges for informative cytokines. The reported concentrations for individual cytokines are highly variable between published studies. This high variation is likely to be primarily because of technical factors in SP processing and storage, use of different quantification methodologies, and a lack of validation of immunoassays to ensure suitability for SP assessment. Because of the large variation between studies, accurate reference ranges cannot yet be determined from the published literature. To progress the clinical utility of SP cytokine analysis will require standardisation and validation of methodologies so that reference ranges for healthy fertile men can be defined.

## AUTHOR CONTRIBUTIONS

*Methodology, investigation, data curation, formal analysis, visualization, writing ‐ original draft*: H.E.L. *Methodology, investigation, data curation, writing ‐ review and editing*: B.M.A. *Conceptualization, methodology, formal analysis, resources, supervision, project administration, funding acquisition, writing ‐ original draft, writing ‐ review and editing*: S.A.R. *Conceptualization, methodology, formal analysis, supervision, funding acquisition, visualization, writing ‐ original draft, writing ‐ review and editing*: D.J.S.

## CONFLICT OF INTEREST STATEMENT

The authors report no conflict of interest.

## Supporting information

Sporting Information

Sporting Information

Sporting Information

## Data Availability

The data supporting the findings of this study were derived from the following resources, which are available in the public domain: PubMed, Web of Science and Scopus.
