# Supplementary material for: Immune regulatory cytokines in seminal plasma of healthy men: A scoping review and analysis of variance
Source: Andrology. 2023 Apr 10;11(7):1245–66. doi: 10.1111/andr.13424 (PMC10947054; doi:10.1111/andr.13424)
Supplement: Supplementary file 1 — Sporting Information [file ANDR-11-1245-s002.pdf]

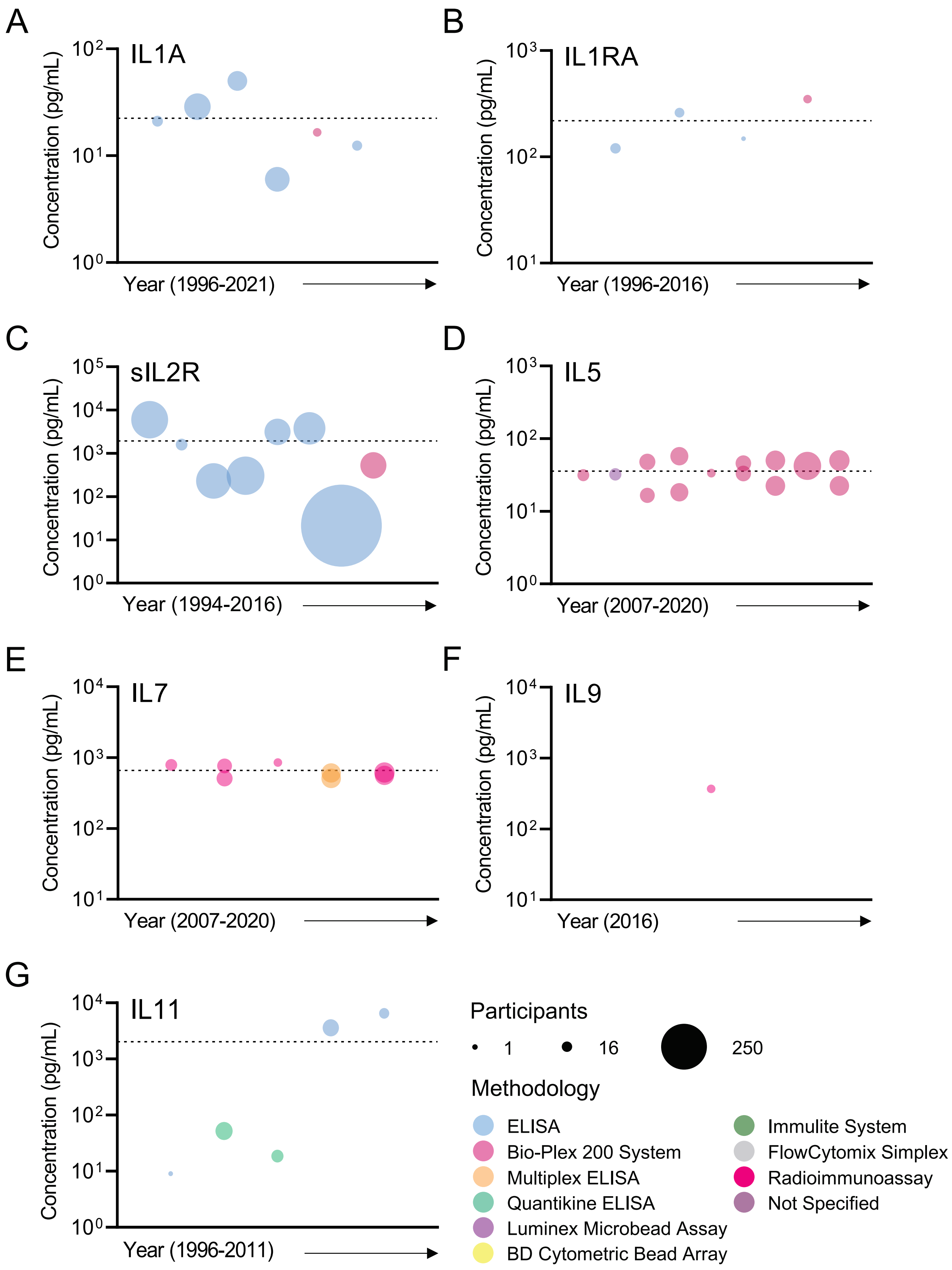

Supplemental Figure S1. Bubble plot graphs representing studies investigating the abundance of IL1A (A), IL1RA (B), sIL2R (C), IL5 (D), IL7 (E), IL9 (F) and IL11 (G) in seminal plasma. Bubbles are ordered along the X-axis from earliest (left) to most recent year (right). Bubbles presented below the horizontal dotted line and ND (Not Detectable, on Y-axis) on individual graphs depict studies reporting the individual cytokine as being undetectable in human seminal plasma. Dashed lines: BLACK = mean concentration of individual cytokine with outliers included, and BLUE = mean cytokine concentration following removal of identified outlier(s).

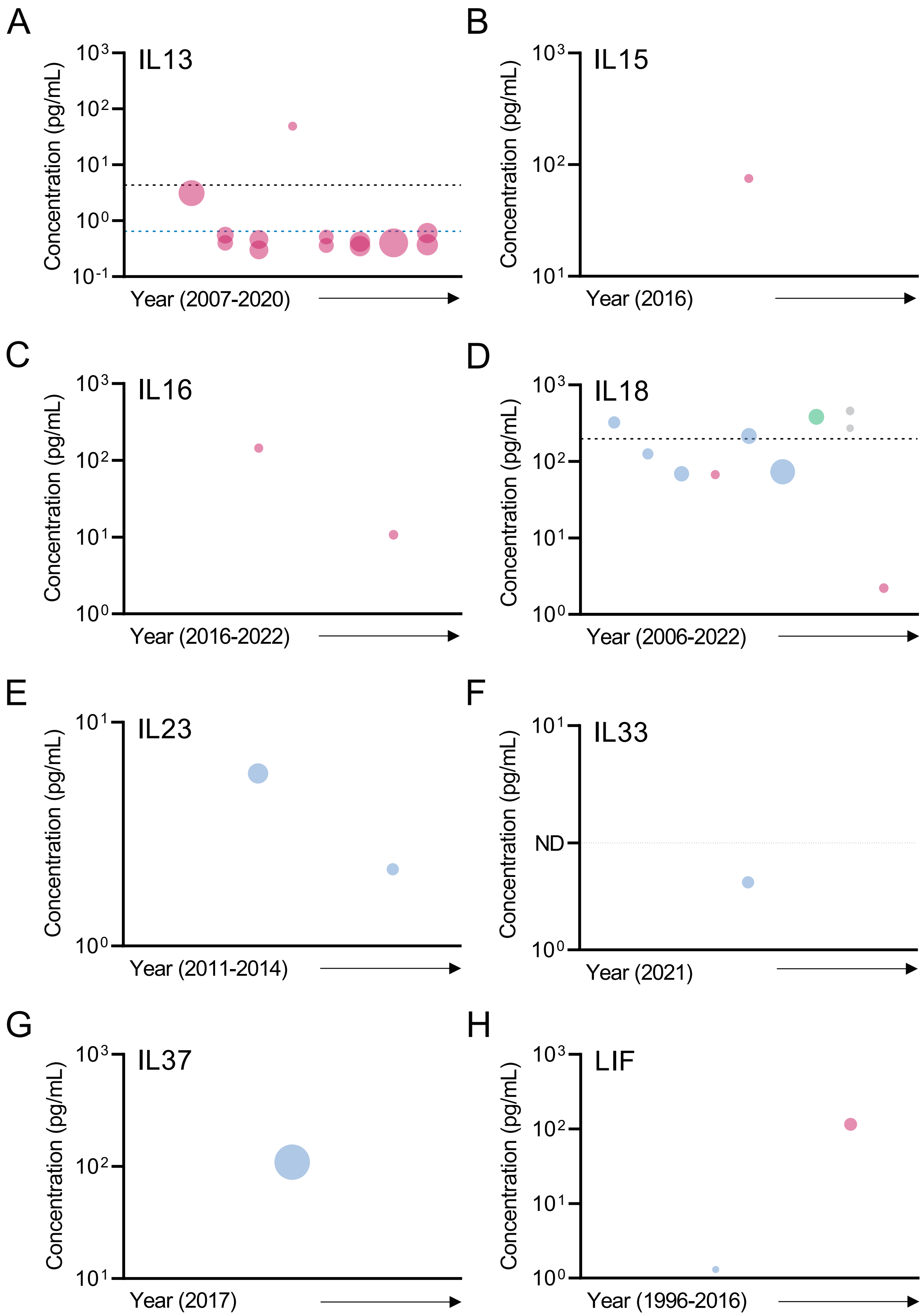

Supplemental Figure S2. Bubble plot graphs visually representing studies investigating the abundance of interleukins in seminal plasma. A detailed legend describing bubble plot size and color as well as annotations is provided within the figure and figure legend text of Supplemental Figure S1.

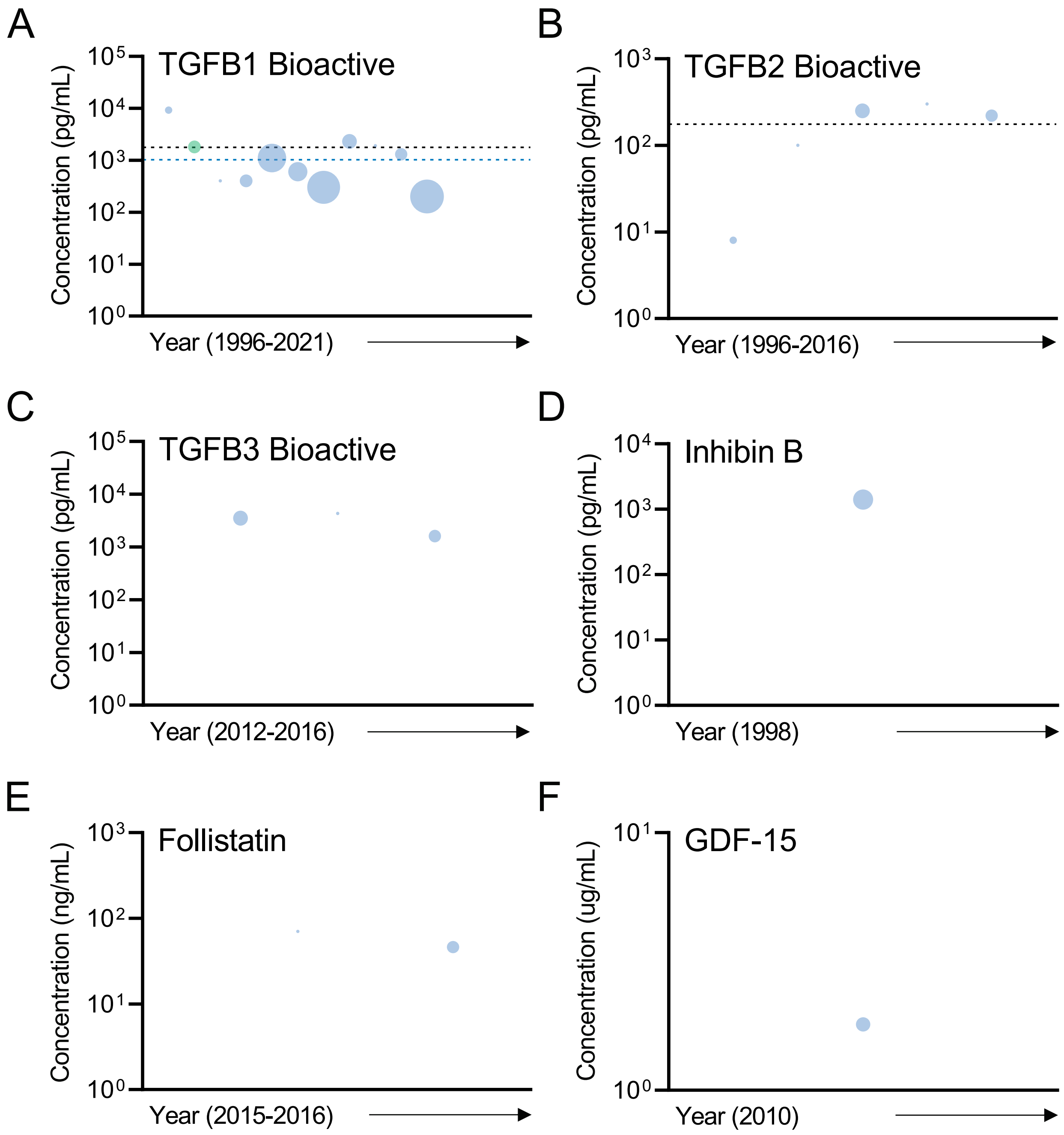

Supplemental Figure S3. Bubble plot graphs visually representing the studies investigating the abundance of the transforming growth factor superfamily in seminal plasma. A detailed legend describing bubble plot size and color as well as annotations is provided within the figure and figure legend text of Supplemental Figure S1.

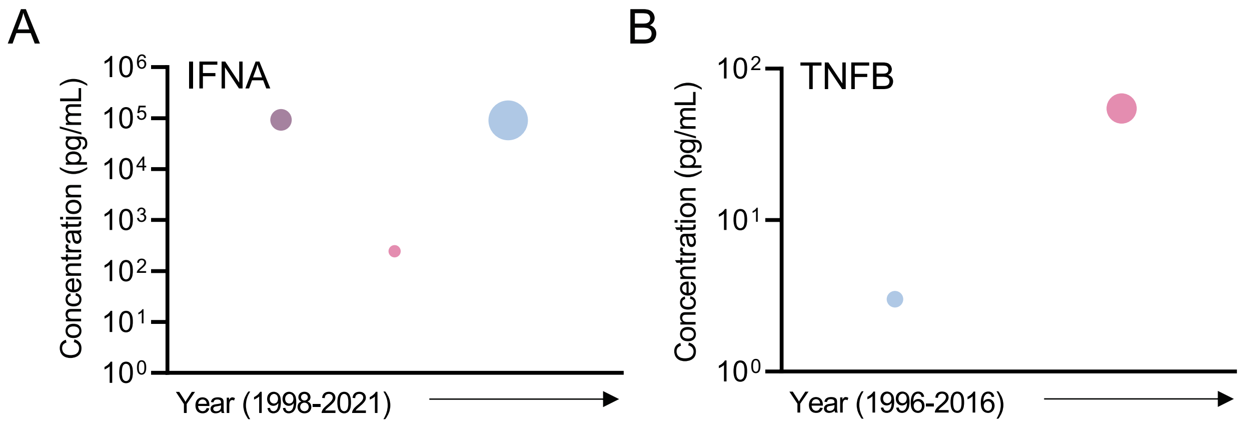

Supplemental Figure S4. Bubble plot graphs visually representing the studies investigating the abundance of interferons, tumor necrosis factors, and colony stimulating factors in seminal plasma. A detailed legend describing bubble plot size and color as well as annotations is provided within the figure and figure legend text of Supplemental Figure S1.

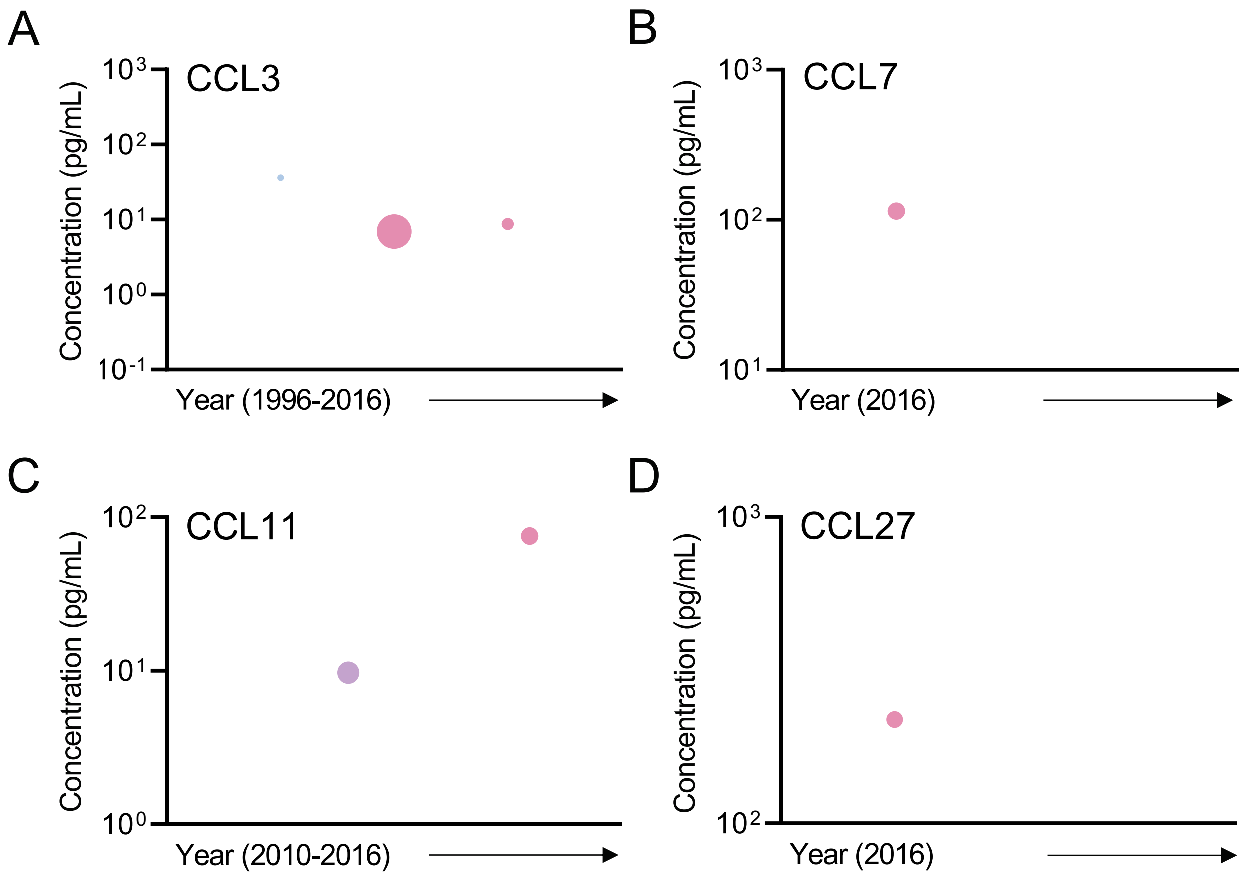

Supplemental Figure S5. Bubble plot graphs visually representing the studies investigating the abundance of (C-C) motif chemokines in seminal plasma. A detailed legend describing bubble plot size and color as well as annotations is provided within the figure and figure legend text of Supplemental Figure S1.

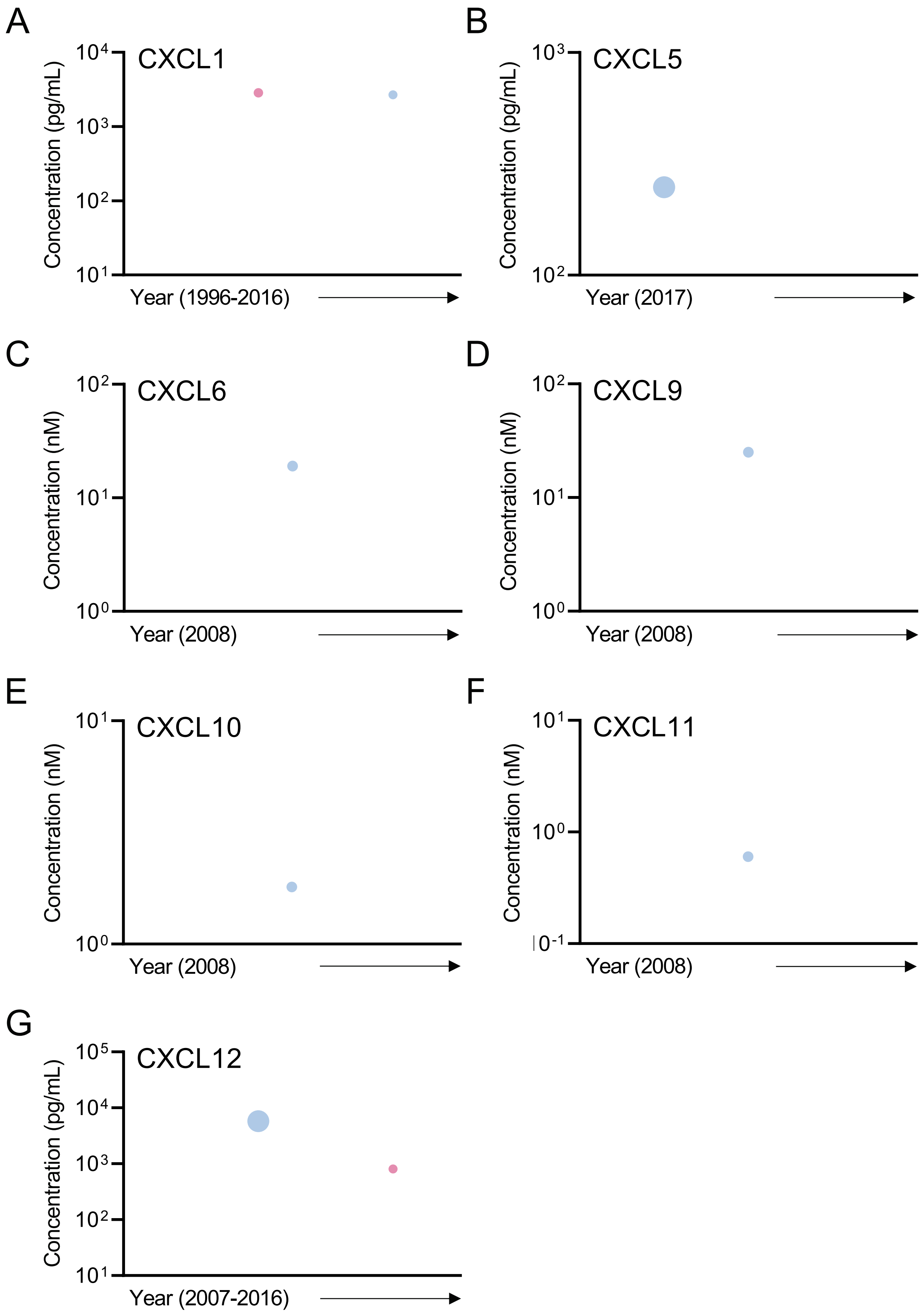

Supplemental Figure S6. Bubble plot graphs visually representing the studies investigating the abundance of (C-X-C) motif chemokines in seminal plasma. A detailed legend describing bubble plot size and color as well as annotations is provided within the figure and figure legend text of Supplemental Figure S1.
