# Supplementary material for: Immune regulatory cytokines in seminal plasma of healthy men: A scoping review and analysis of variance
Source: Andrology. 2023 Apr 10;11(7):1245–66. doi: 10.1111/andr.13424 (PMC10947054; doi:10.1111/andr.13424)
Supplement: Supplementary file 2 — Sporting Information [file ANDR-11-1245-s003.pdf]

Supplemental Table S1: Search strategy employed for PubMed database

(("human"[Text Word] OR "humans"[Text Word] OR "men"[Text Word] OR "man"[Text Word] OR "donor"[Text Word] OR "donors"[Text Word] OR "participant"[Text Word] OR "participants"[Text Word] OR "patient"[Text Word] OR "patients"[Text Word]) AND ("seminal plasma"[Text Word] OR "seminal fluid"[Text Word] OR "semen"[Text Word] OR "ejaculate"[Text Word]) AND ("cytokine"[Text Word] OR "cytokines"[Text Word] OR "chemokine"[Text Word] OR "chemokines"[Text Word] OR "lymphokine"[Text Word] OR "lymphokines"[Text Word] OR "monokine"[Text Word] OR "monokines"[Text Word] OR "interleukin"[Text Word] OR "interleukins"[Text Word] OR "cluster differentiation"[Text Word] OR "cluster of differentiation"[Text Word] OR "growth factor"[Text Word] OR "growth factors"[Text Word] OR "interferon"[Text Word] OR "interferons"[Text Word] OR "transforming growth factor"[Text Word] OR "transforming growth factors"[Text Word] OR "human leukocyte antigen"[Text Word] OR "human leukocyte antigens"[Text Word] OR "prostaglandin"[Text Word] OR "prostaglandins"[Text Word] OR "tumor necrosis factor"[Text Word] OR "tumour necrosis factor"[Text Word])) AND (english[Filter])

Supplemental Table S2: Summary of studies on cytokine abundance and fertility status.

Factor†	Increased in infertility	Decreased in infertility	No change in infertility	Results to date
**Interleukins (IL)**				
IL1A			[22, 23]	Not informative
IL1B	[43, 44]		[10, 15, 45, 46, 47-51]	No consensus
IL1RA	[45]		[22]	No consensus
IL2	[23, 69]		[51, 70]	No consensus
sIL2R	[72]		[5, 43, 71, 73]	Likely not informative
IL4			[51, 74, 75]	Not informative
IL5		[76]		Possible decrease
IL6	[5, 6, 52, 77-82]		[10, 17, 23, 43, 45-47, 50, 85-89]	No consensus
IL7			[60, 76]	Not informative
IL9				N/A
IL10	[86	[78, 101]	[73, 76, 103]	No consensus
IL11	[25]		[86, 88]	No consensus
IL12		[50, 107]	[51, 73, 86, 96, 106]	Likely not informative
IL13			[59, 60]	Not informative
IL15				N/A
IL16				N/A
IL17	[75, 101, 108]		[109	No consensus
IL18	[108]	[7]	[44]	No consensus
IL23	[75, 80]			Possible increase
IL33				N/A
IL37	[111]			Possible increase
LIF				N/A
**Transforming growth factor beta family (TGFB)**				
TGFB1			[47, 102, 118]	Not informative
TGFB2		[121]		Possible decrease
TGFB3		[121]		Possible decrease
Activin A				N/A
Inhibin B				N/A
FST				N/A
GDF-15				N/A
**Interferons (IFN)**				
IFNA	[123]			Possible increase
IFNG	[51, 86, 124]		[52, 123]	No consensus
**Tumor necrosis factors (TNF)**				
TNFA	[86, 101]		[10, 14, 15, 45, 47, 48, 50, 59, 70, 77, 80, 81]	No consensus
TNFB				
TRAIL	[128]			Possible increase
**Colony stimulating factors (CSF)**				
CSF1			[34]	Not informative
CSF2			[59, 60]	Not informative
CSF3			[34, 47]	Not informative
**CC chemokine ligands (CCL)**				
CCL2	[50]		[19, 60]	No consensus
CCL3				N/A
CCL4	[50]		[59, 60]	No consensus
CCL5		[129]		Possible decrease
CCL7				N/A
CCL11				N/A
CCL27				N/A

**C-X-C chemokine ligands (CXCL)**				
CXCL1	[103]			Possible increase
CXCL5	[40]			Possible increase
CXCL6				N/A
CXCL8	[23, 50, 80, 86]		[20, 22, 59, 60, 128]	No consensus
CXCL9				N/A
CXCL10				N/A
CXCL11				N/A
CXCL12				N/A

† data is summarized from 62 studies (of 118 total studies evaluated) that report cytokine abundance in seminal plasma of infertile men, compared to normozoospermic and/or proven fertile men. References to each study are shown in square brackets – refer to reference list in main text for individual publication details. Studies are categorized according to findings of increased abundance, decreased abundance, or no change in abundance of individual cytokines compared to normozoospermic and/or proven fertile men. N/A (not applicable) indicates no studies have evaluated cytokine abundance based on fertility status.

Supplemental Table S3: Summary of studies on cytokine abundance and infection / inflammation status.

Factor†	Increased in infection / inflammation	Decreased in infection / inflammation	No change in infection / inflammation	Results to date
**Interleukins (IL)**				
IL1A	[22] (infect)(L), [34] (L)			Increased in infection and leukocytospermia
IL1B	[8, 10, 15] (infect), [14, 39] (L) (infect) [26] (COVID) [36] (HIV)			Increased in infection and leukocytospermia
IL1RA	[36] (HIV), [22] (L)			Increased in HIV and leukocytospermia
IL2	[16, 21, 23, 68] (infect)			Increased in infection and inflammation
sIL2R				N/A
IL4		[21] (infect)		Decreased in infection
IL5	[36] (HIV)			Increased in HIV
IL6	[8, 10, 23, 75, 82] (inflam/infect) [22, 39, 87, 88] (infect) [26] (COVID) [14, 17, 19, 22, 68, 79, 83, 90] (L)			Increased in infection and inflammation
IL7			[36] (HIV)	No changes with HIV
IL9			[36] (HIV)	No changes with HIV
IL10	[26] (COVID), [102] (L)	[73] (infect)		Increased in Covid-19 and leukocytospermia, decreased in infection
IL11	[23] (infect)			Increased in infection
IL12			[107] (L)	No change in leukocytospermia
IL13				N/A
IL15	[36] (HIV)			Increased in HIV
IL16			[36] (HIV)	No changes in HIV
IL17	[75] (infect) [108] (Hep B)			Increased in infection and inflammation
IL18	[7] (infect) [108] (Hep B)			Increased in infection and inflammation
IL23				N/A
IL33				N/A
IL37				N/A
LIF			[36] (HIV)	No change in HIV
**Transforming growth factor beta family (TGFB)**				
TGFB1			[47, 102, 118] (L)	No change in leukocytospermia
TGFB2				N/A
TGFB3				N/A
Activin A				N/A
Inhibin B				N/A
FST				N/A
GDF-15				N/A
**Interferons (IFN)**				
IFNA	[26] (COVID)			Increased in Covid-19
IFNG	[26] (COVID), [68] (L)		[36] (HIV), [7] (infect) [75] (inflam)	Increased in Covid-19 and leukocytospermia, no change in HIV, genital infection, or inflammation
**Tumor necrosis factors (TNF)**				

TNFA	[10, 68, 83, 89, 127] (infect), [36] (HIV), [26] (COVID), [68, 83, 102, 127] (L)			Increased in infection and inflammation
TNFB				N/A
TRAIL	[75] (infect), [36] (HIV)			Increased in infection and inflammation
**Colony stimulating factors (CSF)**				
CSF1				N/A
CSF2			[36] (HIV)	No change in HIV
CSF3	[34] (L)		[36] (HIV)	No change in HIV, increased in leukocytospermia
**CC chemokine ligands (CCL)**				
CCL2	[19] (L)		[36] (HIV)	No change in HIV, but increased in leukocytospermia
CCL3			[36] (HIV)	No change in HIV
CCL4				N/A
CCL5	[36] (HIV)			Increased in HIV
CCL7			[36] (HIV)	No change in HIV
CCL11			[36] (HIV)	No change in HIV
CCL27			[36] (HIV)	No change in HIV
**C-X-C chemokine ligands (CXCL)**				
CXCL1	[103] (L)		[36] (HIV)	No change in HIV, increased with L
CXCL5				N/A
CXCL6				N/A
CXCL8	[8, 14, 15, 18, 21-23] (infect), [16, 17, 19-22 22] (L) [26] (COVID)			Increased in infection and inflammation
CXCL9				N/A
CXCL10				N/A
CXCL11				N/A
CXCL12				N/A

† data is summarized from 44 studies (of 118 total studies evaluated) that report cytokine abundance in seminal plasma of men with evidence of infection and/or inflammation, compared to normozoospermic and/or proven fertile men. References to each study are shown in square brackets – refer to reference list in main text for individual publication details. Studies are categorized according to findings of increased abundance, decreased abundance, or no change in abundance of individual cytokines compared to normozoospermic and/or proven fertile men. N/A (not applicable) indicates no studies have evaluated cytokine abundance based on fertility status. (HIV) = human immunodeficiency virus infection; (L) = leukocytospermia; (infect) = male accessory gland and/or genital tract infection; (inflam) = inflammation; (Hep B) = hepatitis B infection; (V) = varicocele; (P) = prostatitis; and (COVID) = COVID-19 infection.
