# Supplementary material for: Immune regulatory cytokines in seminal plasma of healthy men: A scoping review and analysis of variance
Source: Andrology. 2023 Apr 10;11(7):1245–66. doi: 10.1111/andr.13424 (PMC10947054; doi:10.1111/andr.13424)
Supplement: Supplementary file 3 — Sporting Information [file ANDR-11-1245-s001.docx]

**Supplemental Table S1:** Search strategy employed for PubMed database

| (("human"[Text Word] OR "humans"[Text Word] OR "men"[Text Word] OR "man"[Text Word] OR "donor"[Text Word] OR "donors"[Text Word] OR "participant"[Text Word] OR "participants"[Text Word] OR "patient"[Text Word] OR "patients"[Text Word]) AND ("seminal plasma"[Text Word] OR "seminal fluid"[Text Word] OR "semen"[Text Word] OR "ejaculate"[Text Word]) AND ("cytokine"[Text Word] OR "cytokines"[Text Word] OR "chemokine"[Text Word] OR "chemokines"[Text Word] OR "lymphokine"[Text Word] OR "lymphokines"[Text Word] OR "monokine"[Text Word] OR "monokines"[Text Word] OR "interleukin"[Text Word] OR "interleukins"[Text Word] OR "cluster differentiation"[Text Word] OR "cluster of differentiation"[Text Word] OR "growth factor"[Text Word] OR "growth factors"[Text Word] OR "interferon"[Text Word] OR "interferons"[Text Word] OR "transforming growth factor"[Text Word] OR "transforming growth factors"[Text Word] OR "human leukocyte antigen"[Text Word] OR "human leukocyte antigens"[Text Word] OR "prostaglandin"[Text Word] OR "prostaglandins"[Text Word] OR "tumor necrosis factor"[Text Word] OR "tumour necrosis factor"[Text Word])) AND (english[Filter]) |
| --- |

**Supplemental Table S2:** Summary of studies on cytokine abundance and fertility status.

| **Factor**^†^ | **Increased in infertility** | **Decreased in infertility** | **No change in infertility** | **Results to date** |
| --- | --- | --- | --- | --- |
| **Interleukins (IL)** | | | | |
| IL1A |  |  | [22, 23] | Not informative |
| IL1B | [43, 44] |  | [10, 15, 45, 46, 47-51] | No consensus |
| IL1RA | [45] |  | [22] | No consensus |
| IL2 | [23, 69] |  | [51, 70] | No consensus |
| sIL2R | [72] |  | [5, 43, 71, 73] | Likely not informative |
| IL4 |  |  | [51, 74, 75] | Not informative |
| IL5 |  | [76] |  | Possible decrease |
| IL6 | [5, 6, 52, 77-82] |  | [10, 17, 23, 43, 45-47, 50, 85-89] | No consensus |
| IL7 |  |  | [60, 76] | Not informative |
| IL9 |  |  |  | N/A |
| IL10 | [86 | [78, 101] | [73, 76, 103] | No consensus |
| IL11 | [25] |  | [86, 88] | No consensus |
| IL12 |  | [50, 107] | [51, 73, 86, 96, 106] | Likely not informative |
| IL13 |  |  | [59, 60] | Not informative |
| IL15 |  |  |  | N/A |
| IL16 |  |  |  | N/A |
| IL17 | [75, 101, 108] |  | [109 | No consensus |
| IL18 | [108] | [7] | [44] | No consensus |
| IL23 | [75, 80] |  |  | Possible increase |
| IL33 |  |  |  | N/A |
| IL37 | [111] |  |  | Possible increase |
| LIF |  |  |  | N/A |
| **Transforming growth factor beta family (TGFB)** | | | | |
| TGFB1 |  |  | [47, 102, 118] | Not informative |
| TGFB2 |  | [121] |  | Possible decrease |
| TGFB3 |  | [121] |  | Possible decrease |
| Activin A |  |  |  | N/A |
| Inhibin B |  |  |  | N/A |
| FST |  |  |  | N/A |
| GDF-15 |  |  |  | N/A |
| **Interferons (IFN)** | | | | |
| IFNA | [123] |  |  | Possible increase |
| IFNG | [51, 86, 127] |  | [52, 123] | No consensus |
| **Tumor necrosis factors (TNF)** | | | | |
| TNFA | [86, 101] |  | [10, 14, 15, 45, 47, 48, 50, 59, 70, 77, 80, 81] | No consensus |
| TNFB |  |  |  |  |
| TRAIL | [131] |  |  | Possible increase |
| **Colony stimulating factors (CSF)** | | | | |
| CSF1 |  |  | [34] | Not informative |
| CSF2 |  |  | [59, 60] | Not informative |
| CSF3 |  |  | [34, 47] | Not informative |
| **CC chemokine ligands (CCL)** | | | | |
| CCL2 | [50] |  | [19, 60] | No consensus |
| CCL3 |  |  |  | N/A |
| CCL4 | [50] |  | [59, 60] | No consensus |
| CCL5 |  | [132] |  | Possible decrease |
| CCL7 |  |  |  | N/A |
| CCL11 |  |  |  | N/A |
| CCL27 |  |  |  | N/A |
| **C-X-C chemokine ligands (CXCL)** | | | | |
| CXCL1 | [103] |  |  | Possible increase |
| CXCL5 | [40] |  |  | Possible increase |
| CXCL6 |  |  |  | N/A |
| CXCL8 | [23, 50, 80, 86] |  | [20, 22, 59, 60, 134] | No consensus |
| CXCL9 |  |  |  | N/A |
| CXCL10 |  |  |  | N/A |
| CXCL11 |  |  |  | N/A |
| CXCL12 |  |  |  | N/A |

† = data is summarized from 62 studies (of 118 total studies evaluated) reporting cytokine abundance in seminal plasma of infertile men, compared to normozoospermic and/or proven fertile men. References to each study are shown in square brackets – refer to reference list in main text for individual publication details. Studies are categorized according to findings of increased abundance, decreased abundance, or no change in abundance of individual cytokines compared to normozoospermic and/or proven fertile men. N/A (not applicable) indicates no studies have evaluated cytokine abundance based on fertility status.

**Supplemental Table S3:** Summary of studies on cytokine abundance and infection / inflammation status.

| **Factor**^†^ | **Increased in infection / inflammation** | **Decreased in infection / inflammation** | **No change in infection / inflammation** | **Results to date** |
| --- | --- | --- | --- | --- |
| **Interleukins (IL)** | | | | |
| IL1A | [22] (infect)(L), [34] (L) |  |  | Increased in infection and leukocytospermia |
| IL1B | [8, 10, 15] (infect), [14, 39] (L) (infect) [26] (COVID) [36] (HIV) |  |  | Increased in infection and leukocytospermia |
| IL1RA | [36] (HIV), [22] (L) |  |  | Increased in HIV and leukocytospermia |
| IL2 | [16, 21, 23, 68] (infect) |  |  | Increased in infection and inflammation |
| sIL2R |  |  |  | N/A |
| IL4 |  | [21] (infect) |  | Decreased in infection |
| IL5 | [36] (HIV) |  |  | Increased in HIV |
| IL6 | [8, 10, 23, 75, 82] (inflam/infect) [22, 39, 87, 88] (infect) [26] (COVID) [14, 17, 19, 22, 68, 79, 83, 90] (L) |  |  | Increased in infection and inflammation |
| IL7 |  |  | [36] (HIV) | No changes with HIV |
| IL9 |  |  | [36] (HIV) | No changes with HIV |
| IL10 | [26] (COVID), [102] (L) | [73] (infect) |  | Increased in Covid-19 and leukocytospermia, decreased in infection |
| IL11 | [23] (infect) |  |  | Increased in infection |
| IL12 |  |  | [107] (L) | No change in leukocytospermia |
| IL13 |  |  |  | N/A |
| IL15 | [36] (HIV) |  |  | Increased in HIV |
| IL16 |  |  | [36] (HIV) | No changes in HIV |
| IL17 | [75] (infect) [108] (Hep B) |  |  | Increased in infection and inflammation |
| IL18 | [7] (infect) [108] (Hep B) |  |  | Increased in infection and inflammation |
| IL23 |  |  |  | N/A |
| IL33 |  |  |  | N/A |
| IL37 |  |  |  | N/A |
| LIF |  |  | [36] (HIV) | No change in HIV |
| **Transforming growth factor beta family (TGFB)** | | | | |
| TGFB1 |  |  | [47, 102, 118] (L) | No change in leukocytospermia |
| TGFB2 |  |  |  | N/A |
| TGFB3 |  |  |  | N/A |
| Activin A |  |  |  | N/A |
| Inhibin B |  |  |  | N/A |
| FST |  |  |  | N/A |
| GDF-15 |  |  |  | N/A |
| **Interferons (IFN)** | | | | |
| IFNA | [26] (COVID) |  |  | Increased in Covid-19 |
| IFNG | [26] (COVID), [68] (L) |  | [36] (HIV), [7] (infect) [75] (inflam) | Increased in Covid-19 and leukocytospermia, no change in HIV, genital infection, or inflammation |
| **Tumor necrosis factors (TNF)** | | | | |
| TNFA | [10, 68, 83, 89, 130] (infect), [36] (HIV), [26] (COVID), [68, 83, 102, 130] (L) |  |  | Increased in infection and inflammation |
| TNFB |  |  |  | N/A |
| TRAIL | [75] (infect), [36] (HIV) |  |  | Increased in infection and inflammation |
| **Colony stimulating factors (CSF)** | | | | |
| CSF1 |  |  |  | N/A |
| CSF2 |  |  | [36] (HIV) | No change in HIV |
| CSF3 | [34] (L) |  | [36] (HIV) | No change in HIV, increased in leukocytospermia |
| **CC chemokine ligands (CCL)** | | | | |
| CCL2 | [19] (L) |  | [36] (HIV) | No change in HIV, but increased in leukocytospermia |
| CCL3 |  |  | [36] (HIV) | No change in HIV |
| CCL4 |  |  |  | N/A |
| CCL5 | [36] (HIV) |  |  | Increased in HIV |
| CCL7 |  |  | [36] (HIV) | No change in HIV |
| CCL11 |  |  | [36] (HIV) | No change in HIV |
| CCL27 |  |  | [36] (HIV) | No change in HIV |
| **C-X-C chemokine ligands (CXCL)** | | | | |
| CXCL1 | [103] (L) |  | [36] (HIV) | No change in HIV, increased with L |
| CXCL5 |  |  |  | N/A |
| CXCL6 |  |  |  | N/A |
| CXCL8 | [8, 14, 15, 18, 21-23] (infect), [16, 17, 19-22 22] (L) [26] (COVID) |  |  | Increased in infection and inflammation |
| CXCL9 |  |  |  | N/A |
| CXCL10 |  |  |  | N/A |
| CXCL11 |  |  |  | N/A |
| CXCL12 |  |  |  | N/A |

† = data is summarized from 44 studies (of 118 total studies evaluated) reporting cytokine abundance in seminal plasma of men with evidence of infection and/or inflammation, compared to normozoospermic and/or proven fertile men. References to each study are shown in square brackets – refer to reference list in main text for individual publication details. Studies are categorized according to findings of increased abundance, decreased abundance, or no change in abundance of individual cytokines compared to normozoospermic and/or proven fertile men. N/A (not applicable) indicates no studies have evaluated cytokine abundance based on fertility status. (HIV) = human immunodeficiency virus infection; (L) = leukocytospermia; (infect) = male accessory gland and/or genital tract infection; (inflam) inflammation; (Hep B) = hepatitis B infection; (V) = varicocele; (P) = prostatitis; and (COVID) = Covid-19 infection.
